# Impact of Food Insecurity Resource Navigation for Primary Care Patients with Diabetes and Hypertension: A Matched Cohort Study

**DOI:** 10.1007/s11606-025-09713-1

**Published:** 2025-07-25

**Authors:** Deeksha Gupta, Darin Thomas, Stella Self, Edward A. Frongillo, Alain H. Litwin, J. Alex Ewing, Lynnette Ramos-Gonzalez, A. Caroline Rudisill

**Affiliations:** 1 Department of Health Promotion, Education, and Behavior, Arnold School of Public Health, University of South Carolina, 915 Greene Street, Columbia, SC 29208 USA; 2 Addiction Medicine Center, Prisma Health, 701 Grove Road, Greenville, SC 29605 USA; 3 Department of Epidemiology and Biostatistics, Arnold School of Public Health, University of South Carolina, 915 Greene Street, Columbia, SC 29208 USA; 4 University of South Carolina School of Medicine, 607 Grove Road, Greenville, SC 29605 USA; 5 Data Support Core, Prisma Health, 605 Grove Road, Greenville, SC 29605 USA; 6 Accountable Communities, Prisma Health, 712 Grove Road, Greenville, SC 29605 USA

**Keywords:** Social risk factors, Resource navigator, Chronic diseases, Healthcare charges, Quality of life

## Abstract

**Background:**

Food insecurity contributes to poor health and increased healthcare costs among patients with chronic diseases. Resource navigators can facilitate community-based resource connections, addressing food insecurity, but the impact on healthcare costs and quality of life remains unclear.

**Objective:**

To examine whether food insecurity resource navigation improves clinical outcomes, healthcare charges, and quality of life for patients with diabetes and/or hypertension.

**Design:**

Matched-control quasi-experimental study.

**Patients:**

Patients underwent screening for food insecurity from July 12, 2021, to December 31, 2022, in South Carolina’s largest health system. Eligible patients (18 + years, having food insecurity, and diabetes and/or hypertension) enrolled for resource navigation in three primary care practices. Of 7592 screened patients, 371 (4.89%) were eligible and 236 (3.11%) consented to participation. A propensity-score matched cohort was obtained from practices without the navigator program. Each group included 219 patients, 20 (9.13%) with diabetes, 110 (50.2%) with hypertension, and 89 (40.6%) both.

**Intervention:**

Resource navigator facilitated food-related community resource connections with 6-month follow-up.

**Main Measures:**

Difference-in-difference specifications were used to examine 6-month differences in clinical outcomes (BMI, blood pressure, HbA1c) and healthcare charges (primary, inpatient, emergency department) between patients with and without navigation support. Quality of life changes were assessed for navigator group patients.

**Key Results:**

Among patients with any primary care charges, those in the navigator group had 54.5% (SE = 0.099; *p* = 0.000) greater increase in 6-month charges than controls. Navigator group patients also had improved quality of life (0.345 quality-adjusted life years gained; *p* = 0.014) over 6 months. Emergency department, inpatient charges, and clinical outcomes did not differ between groups.

**Conclusions:**

Food insecurity resource navigation was associated with increased primary care charges and improved quality of life in patients with chronic diseases, highlighting its value in improving patient care. Studies with larger cohorts and extended follow-ups may reveal substantial effects on other patient outcomes.

**Supplementary Information:**

The online version contains supplementary material available at 10.1007/s11606-025-09713-1.

## INTRODUCTION

Food insecurity is a widely recognized social risk factor linked to chronic diseases. In the USA, food insecurity is common in individuals with diabetes (16% vs. 9%)^1^ and hypertension (19% vs. 16.8%)^2^ than those without these conditions. Individuals with food insecurity have limited access to adequate quantity and quality of food, leading to a suboptimal diet and elevated hemoglobin (HbA1c) and blood pressure (BP) levels.^3^ Food insecurity in individuals with diet-related comorbidities is associated with fewer primary care physician (PCP) visits and reduced likelihood of having a usual care source.^4^ Such care disruption limits access to routine services (e.g., eye exams, diabetes education)^5^ and leads to poor medication adherence.^6^ Food insecurity increases the risk of preventable healthcare use, particularly emergency department (ED) and inpatient (IP) visits,^7^ and contributes an additional $4413.61 and $2175.51 (2018 USD), respectively, to annual healthcare costs for individuals with diabetes and hypertension.^8^ Beyond medical care, addressing unmet social needs is essential for improving health outcomes and reducing excessive healthcare spending in individuals with chronic diseases. North Carolina’s Healthy Opportunities Pilots program for Medicaid beneficiaries demonstrated that health-related social needs support (e.g., via food boxes and housing navigation) can lower long-run healthcare spending.^9^

Health systems are screening to identify patients’ social risks^10^ and collaborating with community-based organizations to address unmet needs through electronic medical record (EMR)-embedded tools and platforms.^11,12^ Patients face access barriers, however, due to competing medical issues, non-response, resource unavailability, transportation issues, long wait times, ineligibility, and language/literacy barriers.^13–16^ Providers struggle with facilitating resource connections due to time constraints, heavy caseloads, referral tracking inefficiencies, and insufficient referral follow-up training.^14,17^ A recent review of healthcare setting-based social risk screening and referral processes suggested that referral uptake varies between 3% and 75%, with higher rates when resource organizations contact referred individuals or where access support was available.^18^

In the healthcare context, resource navigators can mitigate patients’ unmet social needs by identifying community-based resources and maintaining relationships with them. Navigators facilitate resource connections through patient education and follow-up, and coordinate clinical care for comprehensive disease management. Studies examining the impact of resource navigator programs on health outcomes, however, provide conflicting evidence.^19–22^ Moreover, research on the relationship between resource navigation and patients’ healthcare use,^22,23^ costs^24^, and quality of life (QOL)^25^ is limited.

To address this gap, we examined a resource navigator program’s impact on clinical outcomes, healthcare charges, and QOL among patients with food insecurity and diabetes and/or hypertension. We hypothesize that resource navigation facilitating food-related resource connections can reduce HbA1c, BP, body mass index (BMI), and healthcare charges, and improve QOL.

## METHODS

### Resource Navigator Program

In July 2021, Prisma Health, South Carolina’s largest non-profit health system (1.5 million + patients annually), implemented a social risk screening and community resource navigator program in three primary care practices in the Upstate (northwest) region. Practices were selected for high diabetes and hypertension caseloads, urban/rural designations (two urban, one rural), and a mix of internal and family medicine specialties. In 2022, 14.5% of South Carolina households had food insecurity (12.8% nationally).^26^ In 2021, of 813,069 Upstate residents, 75.8% were White, 14.6% Black/African-American, 6.5% Hispanic, with 14.2% in poverty (11.4% nationally), and 13.9% uninsured (10.2% nationally).^27^

Selected practices conducted in-person food insecurity screening with Hunger Vital Sign^28^ questions in an Epic module (health system’s EMR platform): “Within the past 12 months, we worried whether our food would run out before we got money to buy more” and “Within the past 12 months, the food we bought just didn’t last and we didn’t have money to buy more”. Responses of “often true” or “sometimes true” on either question indicated a positive screen. Some practices screened for additional social risks (e.g., transportation, utilities, housing). The navigator conducted phone screenings for program-eligible patients not screened for these risks.

Patients aged 18 + years, with positive food insecurity screens, and diabetes and/or hypertension (identified from Epic registries) were eligible for resource navigation from July 12, 2021, to December 31, 2022. The bilingual (Spanish and English) navigator, experienced in case management and working with community-based organizations, received Epic and screener training. The navigator received screener results within Epic and contacted eligible patients via phone for verbal consent, also offering consent forms by mail or electronically. Upon consent, the navigator provided information on local resources (e.g., food pantries, food banks, utilities support services) via text, email, or mail. Patients on the Supplemental Nutrition Assistance Program were informed about additional resources, and non-enrolled patients were referred to enrollment assistance. Prior food insecurity programs referred individuals to similar resources,^29,30^ including produce prescription and Special Supplemental Nutrition Assistance Program for Women, Infants and Children.^31^ The navigator discussed challenges and strategies to overcome resource access barriers. Follow-up calls occurred every 2–3 weeks for 6 months to address any barriers or new needs, with patients also able to initiate contact. If contact failed, up to five attempts were made (including to the next-of-kin, if necessary) before deeming a patient lost to follow-up.

### Study Population

Of 7592 screened patients, 371 (4.89%) were program-eligible and 236 (3.11%) consented to participation. Controls were matched from practices where the navigator program was unavailable via 1:1 propensity scores on patients’ age, gender, race/ethnicity, diabetes/hypertension status, BMI, primary payer, food insecurity status, screening date, and screening practice designation (family/internal medicine).

### Variables

We examined seven outcomes: (1) HbA1c for diabetes patients, (2) BP for hypertension patients, (3) BMI, (4) PCP (excluding intervention costs) (5) IP, (6) ED charges, and (7) QOL. The EMR-based clinical measures (HbA1C, BP, BMI) were recorded during patient visits. Healthcare charges from Epic included amounts billed by the health system for services during a visit (e.g., treatment, physician time, room and board, supplies, overheads). Patients were assigned zero charges if no visits occurred during the study. Outcomes were assessed at baseline and at 6-month follow-up, defined within an 8-week window before and after the study timepoint. “Baseline” data included the closest available data to the screening date within this window, or the nearest data before screening if no suitable baseline data existed. “Follow-up” data were the closest data to the 6-month follow-up within the same 8-week window.

The navigator collected intervention group’s QOL data at baseline, 6- and 12-month follow-ups using the Euroqol 5-Dimensions 5-Levels (EQ-5D-5L) tool.^32^ The tool measures five health dimensions: mobility, self-care, usual activities, pain/discomfort, and anxiety/depression. Responses ranged from “no problems” to “unable to/extreme problems” and were summarized into an EQ-5D-5L score using US valuation weights.^33^ The tool included a Euroqol Visual Analogue Scale (EQ VAS) that measured patients’ perceived health from 0 (worst imaginable health) to 100 (best imaginable health).

Independent variables included EMR-based patient demographics (age, gender, race/ethnicity, language, primary payer, and comorbidities). Comorbidities including cancer (heart, colorectal, prostate, lung, endometrial), chronic pulmonary disease, heart failure, mental health disorder, asthma, Alzheimer’s, depression, and substance use (alcohol and drug use) were defined using Chronic Conditions Data Warehouse ICD-10 codes.^34^ Cerebrovascular disease was defined using Charlson Comorbidity Index ICD-10 codes.^35^ Patients were identified as having these conditions if they had any defining criteria over the study period.

### Statistical Analysis

We examined group differences in baseline clinical outcomes, healthcare charges, screener responses, anxiety levels (measured using Generalized Anxiety Disorder-7), and patient characteristics using paired *t*-tests, McNemar’s chi-squared test (for binary variables), and Cochran-Mantel–Haenszel test (for variables with > 2 categories). Difference-in-difference models with random effects (${\delta }_{i}$) and random errors $\left({\in }_{it}\right)$ compared changes in HbA1c, BP, and BMI (${\mathrm{Y}}_{\mathrm{it}}\mathrm{)}$ over 6 months between intervention and control groups:

$${Y}_{it}={\beta }_{0}+{\beta }_{1}{GROUP}_{i}+{\beta }_{2}{TIME}_{it}+{\beta }_{3} \left({GROUP}_{i}\times {TIME}_{it}\right)+{\beta }_{4}{DEM}_{i}+{\delta }_{i}+{\in }_{it}$$

The key variable, $\left({GROUP}_{i}\times {TIME}_{it}\right)$, was the interaction between the group $\left({GROUP}_{i}\right)$ and time $\left({TIME}_{it}\right)$ indicators. The coefficient, ${\beta }_{3}$, represented the 6-month differences in outcomes between the groups. Other independent variables were a vector of baseline demographics (${\mathrm{DE}}{\mathrm{M}}_{\mathrm{i}}$).

Two-part models with difference-in-difference specifications estimated 6-month differences in healthcare charges (PCP, ED, and IP) between groups, accounting for a large proportion of zero charges in the data.^36^ The first part modeled the likelihood of incurring any healthcare charge (vs. no charge) using a logit specification. The second part estimated 6-month differences in positive healthcare charges between the groups using generalized estimating equations, with error distribution determined from the modified Park test.^37^ An inverse Gaussian distribution with log link was used for PCP and ED charges, and a gamma distribution with log link for IP charges.

Differences in intervention group’s QOL and perceived health at baseline, and at 6- and 12-month follow-up were examined with a paired *t*-tests. Quality of life-adjusted years (QALYs) gained were calculated for this period using the area under the curve method.^38^

This study was approved by the Institutional Review Board at Prisma Health (IRB no. Pro00105924). Matching was done using the “matchit” package (v.4.5.4) in R (v.4.2.0).^39^ Analysis was performed using Stata/MP (v.11).^40^

## RESULTS

### Study Sample

**Figure 1:**
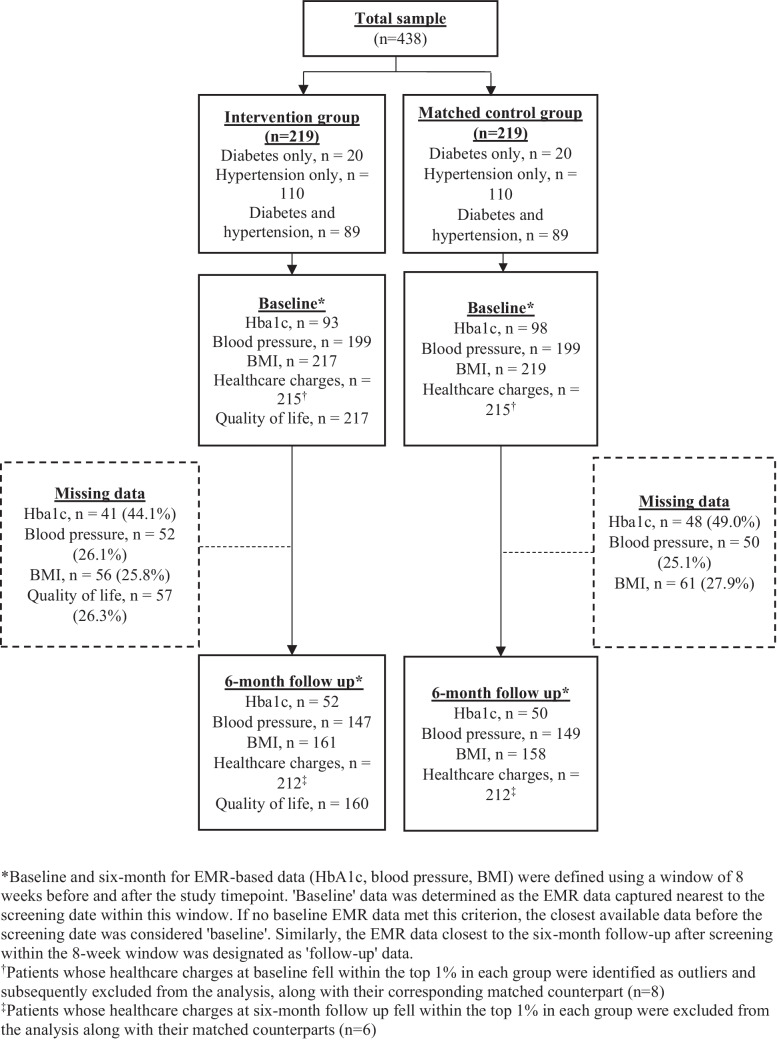
Intervention and control group patients with complete clinical outcomes, healthcare charges, and quality of life data at baseline and the 6-month follow-up.

The study includes 219 patients in each group, with 20 (9.13%) having diabetes, 110 (50.2%) hypertension, and 89 (40.6%) both (Fig. 1). Missing clinical outcomes data were observed at baseline and follow-up. We examined differences in baseline clinical outcomes, screener responses, and demographics between patients with and without missing 6-month follow-up data (Tables A.1–A.3 in Appendix), noting differences in some screener responses, comorbidities, and patient characteristics (e.g., age, payer).

We used Little’s test^41^ to examine whether missingness in clinical outcomes was independent of observed and unobserved variables. Missing baseline and follow-up clinical outcomes data were imputed using multiple imputation (Methods A.1 and Table A.4 in Appendix). The following results include multiply imputed clinical outcomes data.

### Baseline Outcomes and Characteristics

Both groups had similar characteristics, except language, drug abuse disorder, and anxiety levels (Table 1). Compared to controls, fewer intervention group patients preferred English (99.5% vs. 93.6%; *p* = 0.002) and had drug abuse disorder (19.6% vs. 11.4%; *p* = 0.016), but more had moderate to severe anxiety levels (*p* = 0.022). Anxiety data were available for a small subset and may not represent the full sample.

**Table 1:** Clinical Outcomes, Healthcare Charges, and Demographics in Control and Intervention Groups at Baseline

Variable	Control		Intervention		Comparison tests		
	***n* (%)**	**Mean** ** (SD)**	***n* (%)**	**Mean** (SD)	**Diff. mean** (SE)	**Test value** **(df)**	***p***
*Clinical outcomes*							
HbA1c (%)	109	7.28 (1.73)	109	7.72 (2.21)	0.442 (0.256)	1.73	0.084
Systolic BP	199	131.3 (17.5)	199	134.6 (19.2)	3.34 (1.77)	1.88	0.060
Diastolic BP	199	80.8 (11.3)	199	81.6 (14.3)	0.834 (0.950)	0.700	0.48
BMI	219	35.9 (10.5)	219	35.2 (9.72)	− 0.707 (0.967)	− 0.730	0.46
*Healthcare charges (in 2023 USD)*							
PCP charges	212	682.8 (456.3)	212	387.6 (319.6)	− 295.2 (38.2)	− 7.72 (211)	< 0.001
IP charges	212	3782.4 (15,774.4)	212	5877.9 (18,327.9)	2095.5 (1629.5)	1.29 (211)	0.20
ED charges	212	251.4 (628.4)	212	263.4 (571.6)	12.0 (58.5)	0.206 (211)	0.84
*Demographics*							
Age (in years)	219	54.4 (14.4)	219	55.0 (13.8)	− 0.612 (1.14)	− 0.538 (218)	0.59
Exercise duration per week (min)	172	10.9 (11.5)	172	10.0 (11.4)	0.890 (1.19)	0.749 (171)	0.46
Female	154 (70.3%)		144 (65.8%)			1.00 (1)	0.32
English	218 (99.5%)		205 (93.6%)			2 (1)	0.002
Race/ethnicity	219		219			2.99 (4)	0.56
Black/African American	112 (51.1%)		98 (44.8%)				
White	91 (41.6%)		104 (47.5%)				
Hispanic	10 (4.57%)		11 (5.02%)				
Other/more than one race*	5 (2.28%)		4 (1.83%)				
Patient refused/unknown	1 (0.457%)		2 (0.913%)				
Primary payer	219		218			5.08 (5)	0.41
Private/commercial^†^	68 (31.1%)		60 (27.5%)				
Managed Care	34 (15.5%)		25 (11.5%)				
Medicaid^‡^	33 (15.1%)		43 (19.7%)				
Medicare	18 (8.22%)		26 (11.9%)				
Medicare Advantage	36 (16.4%)		32 (14.7%)				
Others^§^	30 (13.7%)		32 (14.7%)				
Comorbidities							
Diabetes only	20 (9.13%)		20 (9.13%)				1.00
Hypertension only	110 (50.2%)		110 (50.2%)				1.00
Diabetes and hypertension	89 (40.6%)		89 (40.6%)				1.00
Cancer	23 (10.5%)		20 (9.13%)			0.243 (1)	0.62
COPD	27 (12.3%)		24 (11.0%)			0.220 (1)	0.64
CHF	22 (10.1%)		26 (11.9%)			0.364 (1)	0.55
CVD	18 (8.22%)		28 (12.8%)			2.38 (1)	0.12
Mental health disorder	91 (41.6%)		82 (37.4%)			0.757 (1)	0.38
Asthma	38 (17.4%)		28 (12.8%)			1.67 (1)	0.20
Alzheimer’s	0 (0.000%)		1 (0.457%)			1.00 (1)	0.32
ETOH abuse	9 (4.11%)		15 (6.85%)			1.50 (1)	0.22
Drug abuse	43 (19.6%)		25 (11.4%)			5.79 (1)	0.016
Anxiety (GAD-7)	31		11			9.67 (3)	0.022
Minimal anxiety	11 (35.5%)		1 (9.09%)				
Mild anxiety	8 (25.8%)		0 (0.000%)				
Moderate anxiety	4 (12.9%)		4 (36.4%)				
Severe anxiety	8 (25.8%)		6 (54.6%)				

*BMI*, body mass index; *BP*, blood pressure; *CHF*, congestive heart failure; *COPD*, chronic obstructive pulmonary disorder; *CVD*, cerebrovascular diseases; *ED*, emergency department; *ETOH*, ethyl alcohol; *IP*, inpatient; *PCP*, primary care physician; *GAD*, general anxiety disorder; *SD*, standard deviation; *SE*, standard error

^*^Other/more than one race includes American Indian or Alaska Native, Native Hawaiian or Other Pacific Islander, Asian, and individuals with two or more races

^†^Private/commercial includes Bluecross Blueshield and Commercial

^‡^Medicaid includes Medicaid and Medicaid MCO

^§^Other payers include other, self-pay, pending Medicaid, and Tricare

Baseline PCP charges were higher for controls than the intervention group (mean[SD] = 682.8[456.3] vs. 387.6[319.6]; *p* < 0.001). Baseline clinical outcomes, IP, and ED charges, and screener responses (Table 2) did not differ between the groups.

**Table 2:** Social Risk Screener Responses in Intervention and Control Groups at Baseline

Variable	Control	Intervention	Comparison tests	
	***n* (%)**	***n* (%)**	**Test value (df)**	***p***
Within the past 12 months, we worried that our food would run out before we got money to buy more	219	219	2.83 (3)	0.42
Never true	16 (7.31%)	15 (6.85%)		
Sometimes true	148 (67.6%)	142 (64.8%)		
Often true	51 (23.3%)	61 (27.9%)		
Patient refused	4 (1.83%)	1 (0.457%)		
Within the past 12 months, the food we bought just did not last and we did not have money to get more	219	219	2.06 (3)	0.56
Never true	30 (13.7%)	31 (14.2%)		
Sometimes true	144 (65.8%)	137 (62.6%)		
Often true	42 (19.2%)	50 (22.8%)		
Patient refused	3 (1.37%)	1 (0.457%)		
How hard is it for you to pay for the very basics like food, housing, medical care, and heating?	218	212	6.32 (5)	0.28
Very hard	31 (14.2%)	40 (18.9%)		
Hard	39 (17.9%)	40 (18.9%)		
Somewhat hard	107 (49.1%)	85 (40.1%)		
Not very hard	27 (12.4%)	35 (16.5%)		
Not hard at all	14 (6.42%)	10 (4.72%)		
Patient refused	0 (0.000%)	2 (0.943%)		
Was there a time in the past 12 months when you needed to see a doctor or buy medications but could not because of cost?	217	211	2.20 (2)	0.33
Yes	87 (40.1%)	101 (47.9%)		
No	127 (58.5%)	107 (50.7%)		
Patient refused	3 (1.38%)	3 (1.42%)		
Do you feel tense, restless, nervous, or anxious most days?	184	209	2.82 (5)	0.73
Very much	32 (17.4%)	42 (20.1%)		
Quite a bit	27 (14.7%)	41 (19.6%)		
To some extent	42 (22.8%)	48 (23.0%)		
Only a little	50 (27.2%)	48 (23.0%)		
Not at all	32 (17.4%)	30 (14.4%)		
Patient refused	1 (0.543%)	0 (0.000%)		
In a typical week, how many times do you talk/text with family, friends, or neighbors?	185	210	6.22 (5)	0.29
More than three times	114 (61.6%)	115 (54.8%)		
Three times a week	21 (11.4%)	28 (13.3%)		
Twice a week	18 (9.73%)	20 (9.52%)		
Once a week	22 (11.9%)	36 (17.1%)		
Never	5 (2.70%)	10 (4.76%)		
Patient refused	5 (2.70%)	1 (0.476%)		
How often do you get together with friends or relatives?	183	208	9.90 (5)	0.078
More than three times	44 (24.0%)	39 (18.8%)		
Three times a week	14 (7.65%)	12 (5.77%)		
Twice a week	24 (13.1%)	36 (17.3%)		
Once a week	68 (37.2%)	81 (38.9%)		
Never	24 (13.1%)	39 (18.8%)		
Patient refused	9 (4.92%)	1 (0.481%)		
In the last 12 months, was there a time when you did not have a steady place to sleep or slept in a shelter?	182	206	0.787 (2)	0.68
Yes	13 (7.14%)	21 (10.2%)		
No	167 (91.8%)	184 (89.3%)		
Patient refused	2 (1.10%)	1 (0.485%)		
Are you worried that the place you are living now is making you sick?	183	205	1.67 (2)	0.43
Yes	18 (9.84%)	15 (7.32%)		
No	159 (86.9%)	187 (91.2%)		
Patient refused	6 (3.28%)	3 (1.46%)		
In the past 12 months, has lack of transportation kept you from medical appointments or medications?	218	214	2.13 (2)	0.35
Yes	44 (20.2%)	47 (22.0%)		
No	174 (79.8%)	165 (77.1%)		
Patient refused	0 (0.000%)	2 (0.934%)		

### Group Differences in Clinical Outcomes and Healthcare Charges

We examined within-group differences in clinical outcomes and healthcare charges at baseline and 6-month follow-up (Table A.5 in Appendix). The intervention group had lower systolic BP (mean[SD] = –5.00[1.55]; *p* = 0.002), and PCP charges (–$154.3[30.4]; *p* < 0.001) at follow-up than baseline. The controls had lower PCP (–$218.9[36.9]; *p* < 0.001) and ED charges (–$92.0[39.2]; *p* = 0.020) over 6 months, but no changes in systolic BP (–1.51[1.63]; *p* = 0.36). No within-group differences in HbA1c, diastolic BP, BMI, and IP charges were observed for either group.

No group differences were observed for clinical outcomes (Table 3; Table A.6 in Appendix for full model): HbA1c (− 0.134[0.230]; *p* = 0.56), systolic (− 3.49[2.20]; *p* = 0.11), and diastolic BP (− 0.664[1.32]; *p* = 0.61), and BMI (− 0.073[0.267]; *p* = 0.79). Two-part models showed no differences in likelihood of any healthcare charges being incurred over 6 months between the groups (Table 4; Table A.7 in Appendix for the full model). Among patients incurring any healthcare charge, however, those receiving navigation had 54.5% (0.545 = exp(0.435) − 1; SE = 0.099; *p* < 0.001) greater PCP charges over 6 months than controls, with no differences in ED (coeff.[SE] = 0.292[0.156]; *p* = 0.061) or IP charges (0.507[0.429]; *p* = 0.24).

**Table 3:** Six-Month Changes in Clinical Outcomes for Patients in Intervention and Control Groups

Variables	(1)	(2)	(3)	(4)
	**HbA1c**	**SBP**	**DBP**	**BMI**
	**Mean (SE)**	**Mean (SE)**	**Mean (SE)**	**Mean (SE)**
Group (ref. = matched control)				
Intervention	0.317 (0.287)	3.11 (1.82)	0.614 (1.20)	0.176 (0.888)
*p-value*	0.27	0.087	0.61	0.84
Time (ref. = base)				
6 months	− 0.160 (0.161)	− 1.51 (1.65)	− 0.892 (0.924)	0.099 (0.190)
*p-value*	0.32	0.36	0.34	0.60
Intervention $\times$ 6 months	− 0.134 (0.230)	− 3.49 (2.20)	− 0.664 (1.32)	− 0.073 (0.267)
*p-value*	0.56	0.11	0.61	0.79
*N*	434	796	796	874
*F*-test^*^	*F* (24, 435.7) = 0.64	*F* (24, 11,712.1) = 67.81	*F* (24, 12,646.4) = 149.08	*F* (24, 236,445.3) = 41.60
Prob > *F*	0.91	< 0.001	< 0.001	< 0.001

Full model results are available in Appendix Table A.6

*BMI*, body mass index; *DBP*, diastolic blood pressure; *SBP*, systolic blood pressure; *SE*, standard error

^*^The *F*-test of overall significance tests the hypothesis whether all the independent variables are jointly significant

**Table 4:** Two-Part Model Depicting 6-Month Changes in Healthcare Charges Between Intervention and Control Groups

Variables	(1)		(2)		(3)	
	**PCP charges**		**ED charges**		**IP charges**	
	First part*, OR	**Second part**†**, Coeff**	First part*, OR	**Second part**†**, Coeff**	First part*, OR	**Second part**‡**, Coeff**
Group (ref. = matched control)						
Intervention	0.101 (0.059)	− 0.425 (0.063)	1.66 (0.527)	− 0.070 (0.138)	1.68 (0.682)	− 0.113 (0.224)
*p-value*	< 0.001	< 0.001	0.11	0.61	0.20	0.62
Time (ref. = base)						
6 months	0.033 (0.020)	− 0.105 (0.060)	0.743 (0.187)	− 0.348 (0.099)	0.681 (0.294)	− 0.292 (0.387)
*p-value*	< 0.001	0.079	0.24	< 0.001	0.37	0.45
Intervention $\times$ 6 months	0.677 (0.388)	0.435 (0.099)	0.700 (0.262)	0.292 (0.156)	0.974 (0.532)	0.507 (0.429)
*p-value*	0.50	< 0.001	0.34	0.061	0.96	0.24
*N*	846	617	846	240	840	71
Wald ${\chi }^{2}$	${\chi }^{2}$(24) = 57.7	${\chi }^{2}$(24) = 316.6	${\chi }^{2}$(24) = 89.2	-	${\chi }^{2}$(23) = 87.5	-
Prob > ${\chi }^{2}$	< 0.001	< 0.001	< 0.001	-	< 0.001	-

Standard error in parenthesis; full model results are available in Appendix Table A.7

*PCP*, primary care physician; *ED*, emergency department; *IP*, inpatient; *OR*, odds ratio

^*^Model includes all healthcare charges and is estimated using a logit model with clustered standard errors

^†^Model includes healthcare charges > 0 and is estimated using generalized estimated equation with an inverse Gaussian distribution, log link function, and robust standard errors

^‡^Model includes healthcare charges > 0 and is estimated using generalized estimating equations with a gamma distribution, log link function, and robust standard errors

For a robustness check, we examined the differences in PCP visits over 6 months between the groups using a generalized negative binomial model (Table A.8 in Appendix). The intervention group had a higher PCP visits rate at follow-up than controls (incidence rate ratio (IRR)[SE] = 1.11[0.050], *p* = 0.039).

### QOL and QALYs Gained

We examined pre-post differences in the intervention group’s EQ-5D-5L responses at baseline, and 6- and 12-month follow-ups (Table A.9 in Appendix). At both follow-ups, fewer patients had severe or extreme anxiety than at baseline (*p* = 0.014), while other health dimensions showed no change.

Intervention group had improved QOL at 6 months, represented by EQ-5D-5L score changes (0.098[0.315]; *p* = 0.014) (Table 5), but not at 12-month follow-up (0.078[0.341]; *p* = 0.071). Patients gained 0.345 and 0.710 QALYs over 6 and 12 months, respectively. Patients’ perceived health (measured from EQ VAS) did not change at 6- (2.24[26.4]; *p* = 0.492) and 12-month follow-up (− 2.03[29.6]; *p* = 0.579).

**Table 5:** EQ-5D-5L Score, Perceived Health, and Quality of Life at Baseline for Patients in the Intervention Group

Variable	*n*	Mean QOL/perceived health (SD)			Mean differences in QOL/perceived health		QALYs gained (area under curve)	
		**Baseline**	**6 months**	**12 months**	**6 months**	**12 months**	**6 months**	**12 months**
					**Mean (SD)**	**Mean (SD)**	**Mean** **(SD)**	**Mean** **(SD)**
EQ-5D-5L score — 6 months*	158	0.643 (0.328)	0.723 (0.280)		0.080 (0.330)		0.342	
*p-value*					0.003			
EQ-5D-5L score — all follow-ups^†^	65	0.641 (0.340)	0.739 (0.253)	0.719 (0.236)	0.098 (0.315)	0.078 (0.341)	0.345	0.710
*p-value*					0.014	0.071		
Predicted EQ-5D-5L score — 6 months^‡^	160	0.667 (0.072)	0.705 (0.072)		0.038 (0.000)		0.343	
*p-value*					< 0.001			
Predicted EQ-5D-5L score— all follow ups^‡^	66	0.667 (0.070)	0.705 (0.070)	0.743 (0.070)	0.038 (0.000)	0.076 (0.000)	0.343	0.696
*p-value*					< 0.001	< 0.001		
Perceived health (EQ VAS) — 6 months	160	58.6 (25.0)	56.9 (25.2)		− 1.71 (30.1)			
*p-value*					0.47			
Perceived health (EQ VAS) — all follow ups	66	59.0 (24.5)	61.3 (22.8)	57.0 (24.2)	2.24 (26.4)	− 2.03 (29.6)		
*p-value*					0.49	0.58		

*EQ-5D-5L*, Euroqol 5-Dimensions 5-Levels; *EQ VAS*, Euroqol Visual Analogue Scale; *QALY*, quality-adjusted life year; *QOL*, quality of life

^*^Excludes 2 patients with missing response for at least one dimension in the EQ-5D-5L survey. Since health values are derived by summing (weighted) responses across all dimension values, health values were not available for these patients

^†^Excludes 1 patient with missing response for at least one dimension in the EQ-5D-5L survey since health values are derived by summing (weighted) responses across all dimensions

^‡^Sensitivity analysis conducted to predict EQ-5D-5L scores for all patients (including those with missing responses) using a mixed linear regression model

The above assessment excluded three patients with incomplete EQ-5D-5L survey responses, precluding score calculation. For sensitivity analysis, we predicted EQ-5D-5L scores for these patients using mixed linear regression.^42^ Independent variables included survey period, patient age, gender, race/ethnicity, and payer. Results showed improved (predicted) QOL at 6- (0.038[0.000]; *p* < 0.001) and 12-month follow-ups (0.076[0.000]; *p* < 0.001], with patients gaining 0.343 and 0.696 QALYs, respectively.

## DISCUSSION

We examined the impact of resource navigation on clinical outcomes, healthcare charges, and QOL for patients with food insecurity and diabetes and/or hypertension. Results show increased PCP charges with navigation among patients incurring any PCP charge but no differences in clinical outcomes or ED or IP charges over 6 months between those with and without navigation support. The navigation group also had improved QOL during this period.

Similar to our study, the Individualized Management for Patient-Centered Targets (IMPaCT) intervention provided community health worker-led navigation to low-income individuals with chronic diseases. Reductions in HbA1c, BP, and BMI were observed over 6^21^ and 9 months^22^, although with *p* > 0.05. Studies with longer follow-ups, however, report improved patient health with navigation. A primary care-based social risk screening and navigation program reduced BP levels over 32–34 months but showed no HbA1c differences between patients with unmet social needs receiving navigation and those without these needs and not receiving navigation services.^43^ A food insecurity screening and navigation program for pregnant women with 40-week follow-ups reported similar findings.^20^ Although the *p*-values for changes in our four clinical outcomes ranged 0.11 to 0.79, the observed decreasing trends could be clinically relevant, suggesting potential benefits to patient care with navigation. Larger sample sizes and longer follow-up periods may be necessary to detect any differences, as seen previously.^20,43^

Research on resource navigation’s impact on healthcare use and costs is limited. A study on Accountable Health Communities’ navigator program for Medicaid and Medicare beneficiaries found no differences in healthcare spending, IP, and ambulatory care admissions between groups with and without navigation support.^24^ However, fewer ED visits were observed among Medicaid beneficiaries receiving navigation. Conversely, IMPaCT studies report fewer hospitalizations and shorter stays with navigation.^22^ Similarly, a telephonic navigation program showed reduced healthcare use over 12 months among high-utilizer patients.^23^ Our results show that among patients who incurred any charges, the navigator group had higher PCP charges over 6 months with no differences in IP and ED charges. The group also had increased PCP visits than controls during this period. Prior research suggests that individuals with diet-related comorbidities (e.g., diabetes and hypertension) and food insecurity have few PCP visits and lack a usual source of care^4^, likely due to tradeoffs between disease management and food-related needs. Existing research has found resource navigation to reduce anxiety^44^ and depression,^25^ improve QOL,^25^ and support ability to seek medical care.^45^ Our observations on QOL improvements and fewer people in the navigator group having anxiety at follow-up suggest that navigation may potentially alleviate patient and potentially food insecurity concerns, enabling patients to prioritize self-health management, including routine PCP/wellness visits, and encourage patient trust and health system use. The lack of a control group for QOL assessment, however, limits our ability to establish causal linkages between resource navigation, QOL, and healthcare use.

Our study demonstrates improved QOL in resource navigation patients. A recent study estimated that interventions eliminating food insecurity could yield 0.008 QALYs gained per person per year.^46^ Few studies, however, have examined the relationship between resource navigation and QOL.^47^ In addition to addressing unmet social needs, navigators can foster a sense of being heard and valued in patients through regular engagement, reducing loneliness^44^ and improving QOL. A study conducted in a socially deprived region in Scotland found no QOL gains over 9 months, although patients with 3 + navigator meetings demonstrated QOL improvements and reduced anxiety and depression,^25^ underscoring the importance of regular engagement. Regular navigator contact can boost a patient’s confidence in engaging with the health system, reducing anxiety and improving their QOL as they become actively involved in primary care, in addition to gaining resource connections through navigation support. Future experimental studies can elaborate on the connections between resource navigation and QOL.

Our study has limitations. First, findings are restricted to primary care practices in a specific region within one health system, restricting generalizability to other populations (e.g., those without healthcare access) or healthcare settings (e.g., emergency or inpatient). Given potentially worse patient health in these settings, greater changes in outcomes could be observed with navigation. Second, although QOL improved in the navigator group, the lack of a control group limits attribution to resource navigation. Additionally, a high rate of missing EQ-5D-5L responses at follow-up posed a challenge in assessing QOL changes across the entire sample. Future studies with experimental designs and higher retention rates could illustrate the mechanisms through which resource navigation affects QOL. Third, baseline HbA1c was well-controlled, considering the recommended range of 7–8%.^48^ Larger differences in outcomes may have emerged with a study of patients with suboptimal glycemic control. Fourth, $\ge$ 25% patients were missing follow-up clinical outcomes data, with differences in characteristics (e.g., age, payers) between those with and without complete data. Nevertheless, Little’s test revealed effective imputation using baseline characteristics. Fifth, our study may be underpowered to detect group differences in outcomes, necessitating larger samples and longer follow-ups. Sixth, < 17% patients completed follow-up food insecurity screening, as they were optional, precluding assessment of the program’s impact on food insecurity. Future studies with consistent follow-up screenings could accurately assess this impact. Finally, we did not analyze program implementation data, though prior research shows program elements (e.g., contact frequency and navigator characteristics) affecting referral uptake and patient outcomes.^18,25,49^ Future investigations should examine how program features affect patient health and navigation’s role in patient care.

Our study demonstrates that food insecurity screening with resource navigation increased PCP charges but did not affect clinical outcomes, ED, and IP charges in patients with chronic diseases. QOL improvements were also observed in patients receiving resource navigation. Our study contributes to the growing research on the effectiveness of social needs programs in clinical settings and can inform health system initiatives to improve patient well-being. Future studies should focus on less-explored outcomes including healthcare costs and QOL and examine how program features affect outcomes.

## Supplementary Information

Below is the link to the electronic supplementary material.

Supplementary file1 (DOCX 163 KB)

## Data Availability

Data are available from Prisma Health but restrictions apply to the availability of these data, which were used under agreement for the current study, and so are not publicly available. De-identified data are however available from the authors upon reasonable request and with permission of Prisma Health.

## Declarations:

### Disclaimer:

The funder has no role in the design and conduct of the study; collection, management, analysis, and interpretation of data; preparation, review, or approval of the manuscript or the decision to submit the manuscript for publication.
