# Supplementary material for: Impact of Food Insecurity Resource Navigation for Primary Care Patients with Diabetes and Hypertension: A Matched Cohort Study
Source: J Gen Intern Med. 2025 Jul 25;41(3):620–30. doi: 10.1007/s11606-025-09713-1 (PMC12960847; doi:10.1007/s11606-025-09713-1)
Supplement: Supplementary file 1 — Supplementary file1 (DOCX 163 KB) [file 11606_2025_9713_MOESM1_ESM.docx]

**Supplementary Information**

**Appendix**

Table A.1: Patient characteristics, social risk screener responses and outcomes for complete vs. missing HbA1c data

Table A.2: Patient characteristics, social risk screener responses and outcomes for complete vs. missing blood pressure (BP) data

Table A.3: Patient characteristics, social risk screener responses and outcomes for complete vs. missing BMI data

Methods A.1: Little’s test for data missing completely at random and multiple imputation

Table A.4: Results from Little’s missing completely at random test for clinical outcomes

Table A.5: Clinical outcomes and healthcare charges for intervention and control group at baseline and six months follow-up

Table A.6: Six-month changes in clinical outcomes for patients in intervention and control group (full model)

Table A.7: Two-part model depicting six-month changes in healthcare charges between intervention and control groups (full model)

Table A.8: Generalized negative binomial model predicting differences in rate of primary care visits at baseline and six-months follow-up between intervention and control groups

Table A.9: Distribution of Euroqol-5D-5L dimension responses at baseline, six- and twelve-month follow-up

**Table A.1: Patient characteristics, social risk screener responses and outcomes at baseline with complete vs. missing HbA1c data at six-month follow-up**

| **Outcomes/ patient characteristics** | | **Missing data** | | | | **Complete data** | | | | **Difference, mean (SE)** | | **Test value**^†^**,**  **t (df)**  **or**  $\boldsymbol{x}^{\boldsymbol{2}}$ **(df)** | | **p** | |
| --- | --- | --- | --- | --- | --- | --- | --- | --- | --- | --- | --- | --- | --- | --- | --- |
|  |  | **n** | | **Mean (SD) or proportion*** | | **n** | | **Mean (SD) or proportion*** | |  |  |  |  |  |  |
| **All patients** | | | | | | | | | | | | | | | |
| HbA1c (%), mean (SD) | | 89 | | 7.60 (2.06) | | 102 | | 7.39 (1.93) | | 0.211 (0.289) | | 0.732 (189) | | 0.465 | |
|  | |  | |  | |  | |  | |  | |  | |  | |
| *Social risk screener responses* | | | |  | |  | |  | |  | |  | |  | |
| Within the past 12 months, we worried that our food would run out before we got money to buy more. | | 116 | |  | | 102 | |  | |  | | 0.917 (3) | | 0.821 | |
|  | Never true | | 10 | | 8.62% | | 7 | | 6.86% | |  |  |  | |  |
|  | Sometimes true | | 76 | | 65.5% | | 63 | | 61.8% | |  |  |  | |  |
|  | Often true | | 29 | | 25.0% | | 31 | | 30.4% | |  |  |  | |  |
|  | Patient refused | | 1 | | 0.862% | | 1 | | 0.980% | |  |  |  | |  |
|  |  | |  | |  | |  | |  | |  |  |  | |  |
| Within the past 12 months, the food we bought just did not last and we did not have money to get more. | | 116 | |  | | 102 | |  | |  | | 1.92 (3) | | 0.589 | |
|  | Never true | | 19 | | 16.4% | | 13 | | 12.8% | |  |  |  | |  |
|  | Sometimes true | | 71 | | 61.2% | | 62 | | 60.8% | |  |  |  | |  |
|  | Often true | | 25 | | 21.6% | | 27 | | 26.5% | |  |  |  | |  |
|  | Patient refused | | 1 | | 0.862% | | 0 | | 0.000% | |  |  |  | |  |
|  |  | |  | |  | |  | |  | |  |  |  | |  |
| How hard is it for you to pay for the very basics like food, housing, medical care, and heating? | | 113 | |  | | 99 | |  | |  | | 4.02 (5) | | 0.546 | |
|  | Very hard | | 21 | | 18.6% | | 22 | | 22.2% | |  |  |  | |  |
|  | Hard | | 26 | | 23.0% | | 15 | | 15.2% | |  |  |  | |  |
|  | Somewhat hard | | 44 | | 38.9% | | 37 | | 37.4% | |  |  |  | |  |
|  | Not very hard | | 15 | | 13.3% | | 18 | | 18.2% | |  |  |  | |  |
|  | Not hard at all | | 6 | | 5.31% | | 7 | | 7.07% | |  |  |  | |  |
|  | Patient refused | | 1 | | 0.885% | | 0 | | 0.000% | |  |  |  | |  |
|  |  | |  | |  | |  | |  | |  |  |  | |  |
| Was there a time in the past 12 months when you needed to see a doctor or buy medications but could not because of cost? | | 111 | |  | | 99 | |  | |  | | 0.345 (2) | | 0.841 | |
|  | Yes | | 47 | | 42.3% | | 40 | | 40.4% | |  |  |  | |  |
|  | No | | 62 | | 55.9% | | 58 | | 58.6% | |  |  |  | |  |
|  | Patient refused | | 2 | | 1.80% | | 1 | | 1.01% | |  |  |  | |  |
|  |  | |  | |  | |  | |  | |  |  |  | |  |
| Do you feel tense, restless, nervous, or anxious most days? | | 97 | |  | | 92 | |  | |  | | 6.53 (4) | | 0.163 | |
|  | Very much | | 19 | | 19.6% | | 15 | | 16.3% | |  |  |  | |  |
|  | Quite a bit | | 16 | | 16.5% | | 17 | | 18.5% | |  |  |  | |  |
|  | To some extent | | 24 | | 24.7% | | 12 | | 13.0% | |  |  |  | |  |
|  | Only a little | | 23 | | 23.7% | | 34 | | 37.0% | |  |  |  | |  |
|  | Not at all | | 15 | | 15.5% | | 14 | | 15.2% | |  |  |  | |  |
|  | Patient refused | | 0 | | 0.000% | | 0 | | 0.000% | |  |  |  | |  |
|  |  | |  | |  | |  | |  | |  |  |  | |  |
| In a typical week, how many times do you talk/text with family, friends, or neighbors? | | 96 | |  | | 92 | |  | |  | | 2.50 (5) | | 0.776 | |
|  | More than three times | | 56 | | 58.3% | | 45 | | 48.9% | |  |  |  | |  |
|  | Three times a week | | 11 | | 11.5% | | 13 | | 14.1% | |  |  |  | |  |
|  | Twice a week | | 10 | | 10.4% | | 11 | | 12.0% | |  |  |  | |  |
|  | Once a week | | 15 | | 15.6% | | 16 | | 17.4% | |  |  |  | |  |
|  | Never | | 3 | | 3.13% | | 4 | | 4.35% | |  |  |  | |  |
|  | Patient refused | | 1 | | 1.04% | | 3 | | 3.26% | |  |  |  | |  |
|  |  | |  | |  | |  | |  | |  |  |  | |  |
| How often do you get together with friends or relatives? | | 95 | |  | | 92 | |  | |  | | 2.35 (5) | | 0.798 | |
|  | More than three times | | 20 | | 21.1% | | 19 | | 20.7% | |  |  |  | |  |
|  | Three times a week | | 9 | | 9.47% | | 6 | | 6.52% | |  |  |  | |  |
|  | Twice a week | | 16 | | 16.8% | | 13 | | 14.1% | |  |  |  | |  |
|  | Once a week | | 37 | | 39.0% | | 36 | | 39.1% | |  |  |  | |  |
|  | Never | | 11 | | 11.6% | | 13 | | 14.1% | |  |  |  | |  |
|  | Patient refused | | 2 | | 2.11% | | 5 | | 5.43% | |  |  |  | |  |
|  |  | |  | |  | |  | |  | |  |  |  | |  |
| In the last 12 months, was there a time when you did not have a steady place to sleep or slept in a shelter? | | 95 | |  | | 90 | |  | |  | | 5.58 (2) | | 0.062 | |
|  | Yes | | 14 | | 14.7% | | 4 | | 4.44% | |  |  |  | |  |
|  | No | | 80 | | 84.2% | | 85 | | 94.4% | |  |  |  | |  |
|  | Patient refused | | 1 | | 1.05% | | 1 | | 1.11% | |  |  |  | |  |
|  |  | |  | |  | |  | |  | |  |  |  | |  |
| Are you worried that the place you are living now is making you sick? | | 95 | |  | | 92 | |  | |  | | 0.644 (2) | | 0.725 | |
|  | Yes | | 8 | | 8.42% | | 5 | | 5.43% | |  |  |  | |  |
|  | No | | 85 | | 89.5% | | 85 | | 92.4% | |  |  |  | |  |
|  | Patient refused | | 2 | | 2.11% | | 2 | | 2.17% | |  |  |  | |  |
|  |  | |  | |  | |  | |  | |  |  |  | |  |
| In the past 12 months, has lack of transportation kept you from medical appointments or medications? | | 114 | |  | |  | | 99 | |  | | 2.74 (2) | | 0.255 | |
|  | Yes | | 29 | | 25.4% | | 16 | | 16.2% | |  |  |  | |  |
|  | No | | 84 | | 73.7% | | 82 | | 82.8% | |  |  |  | |  |
|  | Patient refused | | 1 | | 0.877% | | 1 | | 1.01% | |  |  |  | |  |
|  |  | |  | |  | |  | |  | |  |  |  | |  |
| *Patient characteristics* | |  | |  | |  | |  | |  | |  | |  | |
| Age (in years), mean (SD) | | 116 | | 50.9 (14.6) | | 102 | | 57.0 (13.2) | | **-6.13 (1.90)** | | **-3.23 (216)** | | **0.001** | |
|  |  | |  | |  | |  | |  | |  |  |  | |  |
| Gender | | 116 | |  | | 102 | |  | |  | |  | |  | |
|  | Male | | 37 | | 31.9% | | 34 | | 33.3% | |  | 0.051 (1) | 0.821 | |  |
|  | Female | | 79 | | 68.1% | | 68 | | 66.7% | |  |  |  | |  |
|  |  | |  | |  | |  | |  | |  |  |  | |  |
| Race/ethnicity | | 116 | |  | | 102 | |  | |  | | 4.51 (4) | | 0.342 | |
|  | Hispanic | | 9 | | 7.76% | | 7 | | 6.86% | |  |  |  | |  |
|  | Black/African American | | 50 | | 43.1% | | 45 | | 44.1% | |  |  |  | |  |
|  | Other/more than one race^‡^ | | 7 | | 6.03% | | 1 | | 0.980% | |  |  |  | |  |
|  | Patient refused/unknown | | 1 | | 0.862% | | 2 | | 1.96% | |  |  |  | |  |
|  | White | | 49 | | 42.2% | | 47 | | 46.1% | |  |  |  | |  |
|  |  | |  | |  | |  | |  | |  |  |  | |  |
| Exercise duration per week (in minutes), mean (SD) | | 93 | | 10.3 (12.0) | | 92 | | 9.96 (11.8) | | 0.312 (1.75) | | 0.179 (183) | | 0.858 | |
|  |  | |  | |  | |  | |  | |  |  |  | |  |
| Preferred language | | 116 | |  | | 102 | |  | |  | | 0.280 (1) | | 0.597 | |
|  | Other^§^ | | 5 | | 4.31% | | 6 | | 5.88% | |  |  |  | |  |
|  | English | | 111 | | 95.7% | | 96 | | 94.1% | |  |  |  | |  |
|  |  | |  | |  | |  | |  | |  |  |  | |  |
| Primary payer | |  | |  | |  | |  | |  | | **17.0 (5)** | | **0.004** | |
|  | Private/commercial^\|\|^ | | 20 | | 17.4% | | 33 | | 32.4% | |  |  |  | |  |
|  | Managed Care | | 14 | | 12.2% | | 18 | | 17.7% | |  |  |  | |  |
|  | Medicaid^¶^ | | 26 | | 22.6% | | 9 | | 8.82% | |  |  |  | |  |
|  | Medicare | | 13 | | 11.3% | | 13 | | 12.8% | |  |  |  | |  |
|  | Medicare Advantage | | 18 | | 15.7% | | 19 | | 18.6% | |  |  |  | |  |
|  | Others^#^ | | 24 | | 20.9% | | 10 | | 9.80% | |  |  |  | |  |
|  |  | |  | |  | |  | |  | |  |  |  | |  |
| Presence of comorbidities | | 116 | |  | | 102 | |  | |  | |  | |  | |
|  | Cancer | | 16 | | 13.8% | | 11 | | 10.8% | |  | 0.453 (1) | 0.501 | |  |
|  | COPD | | 11 | | 9.48% | | 15 | | 14.7% | |  | 1.41 (1) | 0.235 | |  |
|  | CHF | | 21 | | 18.1% | | 9 | | 8.82% | |  | **3.94 (1)** | **0.047** | |  |
|  | CVD | | 13 | | 11.2% | | 13 | | 12.8% | |  | 0.122 (1) | 0.727 | |  |
|  | Mental health disorder | | 44 | | 37.9% | | 45 | | 44.1% | |  | 0.860 (1) | 0.354 | |  |
|  | Asthma | | 17 | | 14.7% | | 15 | | 14.7% | |  | 0.000 (1) | 0.992 | |  |
|  | Alzheimer’s | | 1 | | 0.862% | | 0 | | 0.000% | |  | 0.883 (1) | 0.347 | |  |
|  | ETOH abuse | | 7 | | 6.03% | | 3 | | 2.94% | |  | 1.19 (1) | 0.276 | |  |
|  | Drug abuse | | 22 | | 19.0% | | 14 | | 13.7% | |  | 1.08 (1) | 0.298 | |  |
|  |  | |  | |  | |  | |  | |  |  |  | |  |
|  | **Intervention group** | | | | | | | | | | | | | |  |
| HbA1c (%), mean (SD) | | 41 | | 7.95 (2.43) | | 52 | | 7.55 (0.292) | | 0.395 (0.471) | | 0.839 (91) | | 0.404 | |
|  |  | |  | |  | |  | |  | |  |  |  | |  |
| *Social risk screener responses* | | | |  | |  | |  | |  | |  | |  | |
| Within the past 12 months, we worried that our food would run out before we got money to buy more. | | 57 | |  | | 52 | |  | |  | | 2.03 (2) | | 0.362 | |
|  | Never true | | 6 | | 10.5% | | 2 | | 3.85% | |  |  |  | |  |
|  | Sometimes true | | 36 | | 63.2% | | 33 | | 63.5% | |  |  |  | |  |
|  | Often true | | 15 | | 26.3% | | 17 | | 32.7% | |  |  |  | |  |
|  | Patient refused | | 0 | | 0.000% | | 0 | | 0.000% | |  |  |  | |  |
|  |  | |  | |  | |  | |  | |  |  |  | |  |
| Within the past 12 months, the food we bought just did not last and we did not have money to get more. | | 57 | |  | | 52 | |  | |  | | 0.673 (2) | | 0.714 | |
|  | Never true | | 12 | | 21.1% | | 8 | | 15.4% | |  |  |  | |  |
|  | Sometimes true | | 31 | | 54.4% | | 29 | | 55.8% | |  |  |  | |  |
|  | Often true | | 14 | | 24.6% | | 15 | | 28.9% | |  |  |  | |  |
|  | Patient refused | | 0 | | 0.000% | | 0 | | 0.000% | |  |  |  | |  |
|  |  | |  | |  | |  | |  | |  |  |  | |  |
| How hard is it for you to pay for the very basics like food, housing, medical care, and heating? | | 55 | |  | | 49 | |  | |  | | 2.76 (5) | | 0.737 | |
|  | Very hard | | 10 | | 18.2% | | 12 | | 24.5% | |  |  |  | |  |
|  | Hard | | 14 | | 25.5% | | 8 | | 16.3% | |  |  |  | |  |
|  | Somewhat hard | | 17 | | 30.9% | | 18 | | 36.7% | |  |  |  | |  |
|  | Not very hard | | 9 | | 16.4% | | 7 | | 14.3% | |  |  |  | |  |
|  | Not hard at all | | 4 | | 7.27% | | 4 | | 8.16% | |  |  |  | |  |
|  | Patient refused | | 1 | | 1.82% | | 0 | | 0.000% | |  |  |  | |  |
|  |  | |  | |  | |  | |  | |  |  |  | |  |
| Was there a time in the past 12 months when you needed to see a doctor or buy medications but could not because of cost? | | 53 | |  | | 49 | |  | |  | | 0.178 (2) | | 0.915 | |
|  | Yes | | 26 | | 49.1% | | 22 | | 44.9% | |  |  |  | |  |
|  | No | | 26 | | 49.1% | | 26 | | 53.1% | |  |  |  | |  |
|  | Patient refused | | 1 | | 1.89% | | 1 | | 2.04% | |  |  |  | |  |
|  |  | |  | |  | |  | |  | |  |  |  | |  |
| Do you feel tense, restless, nervous, or anxious most days? | | 55 | |  | | 48 | |  | |  | | 2.44 (4) | | 0.655 | |
|  | Very much | | 11 | | 20.0% | | 9 | | 18.8% | |  |  |  | |  |
|  | Quite a bit | | 11 | | 20.0% | | 12 | | 25.0% | |  |  |  | |  |
|  | To some extent | | 10 | | 18.2% | | 9 | | 18.8% | |  |  |  | |  |
|  | Only a little | | 13 | | 23.6% | | 14 | | 29.2% | |  |  |  | |  |
|  | Not at all | | 10 | | 18.2% | | 4 | | 8.33% | |  |  |  | |  |
|  | Patient refused | | 0 | | 0.000% | | 0 | | 0.000% | |  |  |  | |  |
|  |  | |  | |  | |  | |  | |  |  |  | |  |
| In a typical week, how many times do you talk/text with family, friends, or neighbors? | | 54 | |  | | 48 | |  | |  | | 4.42 (5) | | 0.491 | |
|  | More than three times | | 27 | | 50.0% | | 21 | | 43.8% | |  |  |  | |  |
|  | Three times a week | | 8 | | 14.8% | | 5 | | 10.4% | |  |  |  | |  |
|  | Twice a week | | 3 | | 5.56% | | 8 | | 16.7% | |  |  |  | |  |
|  | Once a week | | 12 | | 22.2% | | 11 | | 22.9% | |  |  |  | |  |
|  | Never | | 3 | | 5.56% | | 3 | | 6.25% | |  |  |  | |  |
|  | Patient refused | | 1 | | 1.85% | | 0 | | 0.000% | |  |  |  | |  |
|  |  | |  | |  | |  | |  | |  |  |  | |  |
| How often do you get together with friends or relatives? | | 54 | |  | | 48 | |  | |  | | 5.01 (5) | | 0.415 | |
|  | More than three times | | 9 | | 16.7% | | 11 | | 22.9% | |  |  |  | |  |
|  | Three times a week | | 5 | | 9.26% | | 1 | | 2.08% | |  |  |  | |  |
|  | Twice a week | | 10 | | 18.5% | | 10 | | 20.8% | |  |  |  | |  |
|  | Once a week | | 22 | | 40.7% | | 16 | | 33.3% | |  |  |  | |  |
|  | Never | | 7 | | 13.0% | | 10 | | 20.8% | |  |  |  | |  |
|  | Patient refused | | 1 | | 1.85% | | 0 | | 0.000% | |  |  |  | |  |
|  |  | |  | |  | |  | |  | |  |  |  | |  |
| In the last 12 months, was there a time when you did not have a steady place to sleep or slept in a shelter? | | 54 | |  | | 47 | |  | |  | | 2.90 (2) | | 0.234 | |
|  | Yes | | 8 | | 14.8% | | 3 | | 6.38% | |  |  |  | |  |
|  | No | | 46 | | 85.2% | | 43 | | 91.5% | |  |  |  | |  |
|  | Patient refused | | 0 | | 0.000% | | 1 | | 2.13% | |  |  |  | |  |
|  |  | |  | |  | |  | |  | |  |  |  | |  |
| Are you worried that the place you are living now is making you sick? | | 54 | |  | | 48 | |  | |  | | 2.36 (2) | | 0.307 | |
|  | Yes | | 5 | | 9.26% | | 1 | | 2.08% | |  |  |  | |  |
|  | No | | 48 | | 88.9% | | 46 | | 95.8% | |  |  |  | |  |
|  | Patient refused | | 1 | | 1.85% | | 1 | | 2.08% | |  |  |  | |  |
|  |  | |  | |  | |  | |  | |  |  |  | |  |
| In the past 12 months, has lack of transportation kept you from medical appointments or medications? | | 56 | |  | | 49 | |  | |  | | 1.50 (2) | | 0.472 | |
|  | Yes | | 16 | | 28.6% | | 9 | | 18.4% | |  |  |  | |  |
|  | No | | 39 | | 69.6% | | 39 | | 79.6% | |  |  |  | |  |
|  | Patient refused | | 1 | | 1.79% | | 1 | | 2.04% | |  |  |  | |  |
|  |  | |  | |  | |  | |  | |  |  |  | |  |
| *Patient characteristics* | |  | |  | |  | |  | |  | |  | |  | |
| Age | | 57 | | 50.6 (14.8) | | 52 | | 57.2 (12.7) | | **-6.63 (2.65)** | | **-2.50 (107)** | | **0.014** | |
|  |  | |  | |  | |  | |  | |  |  |  | |  |
| Gender | | 57 | |  | | 52 | |  | |  | | 0.049 (1) | | 0.825 | |
|  | Male | | 22 | | 38.6% | | 19 | | 36.5% | |  |  |  | |  |
|  | Female | | 35 | | 61.4% | | 33 | | 63.5% | |  |  |  | |  |
|  |  | |  | |  | |  | |  | |  |  |  | |  |
| Race/ethnicity | | 57 | |  | | 52 | |  | |  | | 2.82 (4) | | 0.589 | |
|  | Hispanic | | 4 | | 7.02% | | 4 | | 7.69% | |  |  |  | |  |
|  | Black/African American | | 22 | | 38.6% | | 21 | | 40.4% | |  |  |  | |  |
|  | Other/more than one race^‡^ | | 3 | | 5.26% | | 0 | | 0.000% | |  |  |  | |  |
|  | Patient refused/unknown | | 1 | | 1.75% | | 1 | | 1.92% | |  |  |  | |  |
|  | White | | 27 | | 47.4% | | 26 | | 50.0% | |  |  |  | |  |
|  |  | |  | |  | |  | |  | |  |  |  | |  |
| Exercise duration per week (in minutes), mean (SD) | | 54 | | 7.74 (10.8) | | 48 | | 10.3 (12.4) | | -2.55 (2.29) | | -1.11 (100) | | 0.268 | |
|  |  | |  | |  | |  | |  | |  |  |  | |  |
| Preferred language | | 57 | |  | | 52 | |  | |  | | 0.023 (1) | | 0.879 | |
|  | Other^§^ | | 5 | | 8.77% | | 5 | | 9.62% | |  |  |  | |  |
|  | English | | 52 | | 91.2% | | 47 | | 90.4% | |  |  |  | |  |
|  |  | |  | |  | |  | |  | |  |  |  | |  |
| Primary payer | | 56 | |  | | 52 | |  | |  | | 10.2 (5) | | 0.070 | |
|  | Private/commercial^\|\|^ | | 9 | | 16.1% | | 20 | | 38.5% | |  |  |  | |  |
|  | Managed Care | | 6 | | 10.7% | | 7 | | 13.5% | |  |  |  | |  |
|  | Medicaid^¶^ | | 16 | | 28.6% | | 6 | | 11.5% | |  |  |  | |  |
|  | Medicare | | 6 | | 10.7% | | 6 | | 11.5% | |  |  |  | |  |
|  | Medicare Advantage | | 8 | | 14.3% | | 7 | | 13.5% | |  |  |  | |  |
|  | Others^#^ | | 11 | | 19.6% | | 6 | | 11.5% | |  |  |  | |  |
|  |  | |  | |  | |  | |  | |  |  |  | |  |
| Presence of comorbidities | | 57 | |  | | 52 | |  | |  | |  | |  | |
|  | Cancer | | 7 | | 12.3% | | 6 | | 11.5% | |  | 0.014 (1) | 0.905 | |  |
|  | COPD | | 6 | | 10.5% | | 7 | | 13.5% | |  | 0.223 (1) | 0.637 | |  |
|  | CHF | | 12 | | 21.1% | | 4 | | 7.69% | |  | **3.88 (1)** | **0.049** | |  |
|  | CVD | | 8 | | 14.0% | | 8 | | 15.4% | |  | 0.040 (1) | 0.842 | |  |
|  | Mental health disorder | | 16 | | 28.1% | | 24 | | 46.2% | |  | 3.83 (1) | 0.050 | |  |
|  | Asthma | | 6 | | 10.5% | | 8 | | 15.4% | |  | 0.573 (1) | 0.449 | |  |
|  | Alzheimer’s | | 1 | | 1.75% | | 0 | | 0.000% | |  | 0.921 (1) | 0.337 | |  |
|  | ETOH abuse | | 4 | | 7.02% | | 3 | | 5.77% | |  | 0.071 (1) | 0.791 | |  |
|  | Drug abuse | | 8 | | 14.0% | | 7 | | 13.5% | |  | 0.008 (1) | 0.931 | |  |
|  |  | |  | |  | |  | |  | |  |  |  | |  |
|  | **Control group** | | | | | | | | | | | | | |  |
| HbA1c (%), mean (SD) | | 48 | | 7.30 (1.67) | | 50 | | 7.21 (1.73) | | 0.086 (0.343) | | 0.251 (96) | | 0.803 | |
|  |  | |  | |  | |  | |  | |  |  |  | |  |
| *Social risk screener responses* | | | |  | |  | |  | |  | |  | |  | |
| Within the past 12 months, we worried that our food would run out before we got money to buy more. | | 59 | |  | | 50 | |  | |  | | 0.802 (3) | | 0.849 | |
|  | Never true | | 4 | | 6.78% | | 5 | | 10.0% | |  |  |  | |  |
|  | Sometimes true | | 40 | | 67.8% | | 30 | | 60.0% | |  |  |  | |  |
|  | Often true | | 14 | | 23.7% | | 14 | | 28.0% | |  |  |  | |  |
|  | Patient refused | | 1 | | 1.69% | | 1 | | 2.00% | |  |  |  | |  |
|  |  | |  | |  | |  | |  | |  |  |  | |  |
| Within the past 12 months, the food we bought just did not last and we did not have money to get more. | | 59 | |  | | 50 | |  | |  | | 1.31 (3) | | 0.726 | |
|  | Never true | | 7 | | 11.9% | | 5 | | 10.0% | |  |  |  | |  |
|  | Sometimes true | | 40 | | 67.8% | | 33 | | 66.0% | |  |  |  | |  |
|  | Often true | | 11 | | 18.6% | | 12 | | 24.0% | |  |  |  | |  |
|  | Patient refused | | 1 | | 1.69% | | 0 | | 0.000% | |  |  |  | |  |
|  |  | |  | |  | |  | |  | |  |  |  | |  |
| How hard is it for you to pay for the very basics like food, housing, medical care, and heating? | | 58 | |  | | 50 | |  | |  | | 3.85 (4) | | 0.426 | |
|  | Very hard | | 11 | | 19.0% | | 10 | | 20.0% | |  |  |  | |  |
|  | Hard | | 12 | | 20.7% | | 7 | | 14.0% | |  |  |  | |  |
|  | Somewhat hard | | 27 | | 46.6% | | 19 | | 38.0% | |  |  |  | |  |
|  | Not very hard | | 6 | | 10.3% | | 11 | | 22.0% | |  |  |  | |  |
|  | Not hard at all | | 2 | | 3.45% | | 3 | | 6.00% | |  |  |  | |  |
|  | Patient refused | | 0 | | 0.000% | | 0 | | 0.000% | |  |  |  | |  |
|  |  | |  | |  | |  | |  | |  |  |  | |  |
| Was there a time in the past 12 months when you needed to see a doctor or buy medications but could not because of cost? | | 58 | |  | | 50 | |  | |  | | 0.878 (2) | | 0.645 | |
|  | Yes | | 21 | | 36.2% | | 18 | | 36.0% | |  |  |  | |  |
|  | No | | 36 | | 62.1% | | 32 | | 64.0% | |  |  |  | |  |
|  | Patient refused | | 1 | | 1.72% | | 0 | | 0.000% | |  |  |  | |  |
|  |  | |  | |  | |  | |  | |  |  |  | |  |
| Do you feel tense, restless, nervous, or anxious most days? | | 42 | |  | | 44 | |  | |  | | **12.4 (4)** | | **0.015** | |
|  | Very much | | 8 | | 19.1% | | 6 | | 13.6% | |  |  |  | |  |
|  | Quite a bit | | 5 | | 11.9% | | 5 | | 11.4% | |  |  |  | |  |
|  | To some extent | | 14 | | 33.3% | | 3 | | 6.82% | |  |  |  | |  |
|  | Only a little | | 10 | | 23.8% | | 20 | | 45.5% | |  |  |  | |  |
|  | Not at all | | 5 | | 11.9% | | 10 | | 22.7% | |  |  |  | |  |
|  | Patient refused | | 0 | | 0.000% | | 0 | | 0.000% | |  |  |  | |  |
|  |  | |  | |  | |  | |  | |  |  |  | |  |
| In a typical week, how many times do you talk/text with family, friends, or neighbors? | | 42 | |  | | 44 | |  | |  | | 8.80 (5) | | 0.117 | |
|  | More than three times | | 29 | | 69.1% | | 24 | | 54.6% | |  |  |  | |  |
|  | Three times a week | | 3 | | 7.14% | | 8 | | 18.2% | |  |  |  | |  |
|  | Twice a week | | 7 | | 16.7% | | 3 | | 6.82% | |  |  |  | |  |
|  | Once a week | | 3 | | 7.14% | | 5 | | 11.4% | |  |  |  | |  |
|  | Never | | 0 | | 0.000% | | 1 | | 2.27% | |  |  |  | |  |
|  | Patient refused | | 0 | | 0.000% | | 3 | | 6.82% | |  |  |  | |  |
|  |  | |  | |  | |  | |  | |  |  |  | |  |
| How often do you get together with friends or relatives? | | 41 | |  | | 44 | |  | |  | | 5.01 (5) | | 0.415 | |
|  | More than three times | | 11 | | 26.8% | | 8 | | 18.2% | |  |  |  | |  |
|  | Three times a week | | 4 | | 9.76% | | 5 | | 11.4% | |  |  |  | |  |
|  | Twice a week | | 6 | | 14.6% | | 3 | | 6.82% | |  |  |  | |  |
|  | Once a week | | 15 | | 36.6% | | 20 | | 45.5% | |  |  |  | |  |
|  | Never | | 4 | | 9.76% | | 3 | | 6.82% | |  |  |  | |  |
|  | Patient refused | | 1 | | 2.44% | | 5 | | 11.4% | |  |  |  | |  |
|  |  | |  | |  | |  | |  | |  |  |  | |  |
| In the last 12 months, was there a time when you did not have a steady place to sleep or slept in a shelter? | | 41 | |  | | 43 | |  | |  | | 5.37 (2) | | 0.068 | |
|  | Yes | | 6 | | 14.6% | | 1 | | 2.33% | |  |  |  | |  |
|  | No | | 34 | | 82.9% | | 42 | | 97.7% | |  |  |  | |  |
|  | Patient refused | | 1 | | 2.44% | | 0 | | 0.000% | |  |  |  | |  |
|  |  | |  | |  | |  | |  | |  |  |  | |  |
| Are you worried that the place you are living now is making you sick? | | 41 | |  | | 44 | |  | |  | | 0.090 (2) | | 0.956 | |
|  | Yes | | 3 | | 7.32% | | 4 | | 9.09% | |  |  |  | |  |
|  | No | | 37 | | 90.2% | | 39 | | 88.6% | |  |  |  | |  |
|  | Patient refused | | 1 | | 2.44% | | 1 | | 2.27% | |  |  |  | |  |
|  |  | |  | |  | |  | |  | |  |  |  | |  |
| In the past 12 months, has lack of transportation kept you from medical appointments or medications? | | 58 | |  | | 50 | |  | |  | | 1.26 (1) | | 0.262 | |
|  | Yes | | 13 | | 22.4% | | 7 | | 14.0% | |  |  |  | |  |
|  | No | | 45 | | 77.6% | | 43 | | 86.0% | |  |  |  | |  |
|  | Patient refused | | 0 | | 0.000% | | 0 | | 0.000% | |  |  |  | |  |
|  |  | |  | |  | |  | |  | |  |  |  | |  |
| *Patient characteristics* | |  | |  | |  | |  | |  | |  | |  | |
| Age (in years), mean (SD) | | 59 | | 51.3 (14.7) | | 50 | | 56.9 (13.7) | | **-5.63 (2.74)** | | **-2.05 (107)** | | **0.042** | |
|  |  | |  | |  | |  | |  | |  |  |  | |  |
| Gender | | 59 | |  | | 50 | |  | |  | | 0.284 (1) | | 0.594 | |
|  | Male | | 15 | | 25.4% | | 15 | | 30.0% | |  |  |  | |  |
|  | Female | | 44 | | 74.6% | | 35 | | 70.0% | |  |  |  | |  |
|  |  | |  | |  | |  | |  | |  |  |  | |  |
| Race/ethnicity | | 59 | |  | | 50 | |  | |  | | 2.91 (4) | | 0.573 | |
|  | Hispanic | | 5 | | 8.47% | | 3 | | 6.00% | |  |  |  | |  |
|  | Black/African American | | 28 | | 47.5% | | 24 | | 48.0% | |  |  |  | |  |
|  | Other/more than one race^‡^ | | 4 | | 6.78% | | 1 | | 2.00% | |  |  |  | |  |
|  | Patient refused/unknown | | 0 | | 0.000% | | 1 | | 2.00% | |  |  |  | |  |
|  | White | | 22 | | 37.3% | | 21 | | 42.0% | |  |  |  | |  |
|  |  | |  | |  | |  | |  | |  |  |  | |  |
| Exercise duration per week (in minutes), mean (SD) | | 39 | | 13.8 (12.8) | | 44 | | 9.59 (11.3) | | 4.18 (2.64) | | 1.58 (81) | | 0.118 | |
|  |  | |  | |  | |  | |  | |  |  |  | |  |
| Preferred language | | 59 | |  | | 50 | |  | |  | | 1.19 (1) | | 0.275 | |
|  | Other^§^ | | 0 | | 0.000% | | 1 | | 2.00% | |  |  |  | |  |
|  | English | | 59 | | 100% | | 49 | | 98.0% | |  |  |  | |  |
|  |  | |  | |  | |  | |  | |  |  |  | |  |
| Primary payer | | 59 | |  | | 50 | |  | |  | | 8.67 (5) | | 0.123 | |
|  | Private/commercial^\|\|^ | | 11 | | 18.6% | | 13 | | 26.0% | |  |  |  | |  |
|  | Managed Care | | 8 | | 13.6% | | 11 | | 22.0% | |  |  |  | |  |
|  | Medicaid^¶^ | | 10 | | 17.0% | | 3 | | 6.00% | |  |  |  | |  |
|  | Medicare | | 7 | | 11.9% | | 7 | | 14.0% | |  |  |  | |  |
|  | Medicare Advantage | | 10 | | 17.0% | | 12 | | 24.0% | |  |  |  | |  |
|  | Others^#^ | | 13 | | 22.0% | | 4 | | 8.00% | |  |  |  | |  |
|  |  | |  | |  | |  | |  | |  |  |  | |  |
| Presence of comorbidities | | 59 | |  | | 50 | |  | |  | |  | |  | |
|  | Cancer | | 9 | | 15.3% | | 5 | | 10.0% | |  | 0.667 (1) | 0.414 | |  |
|  | COPD | | 5 | | 8.47% | | 8 | | 16.0% | |  | 1.46 (1) | 0.227 | |  |
|  | CHF | | 9 | | 15.3% | | 5 | | 10.0% | |  | 0.667 (1) | 0.414 | |  |
|  | CVD | | 5 | | 8.47% | | 5 | | 10.0% | |  | 0.076 (1) | 0.783 | |  |
|  | Mental health disorder | | 28 | | 47.5% | | 21 | | 42.0% | |  | 0.326 (1) | 0.568 | |  |
|  | Asthma | | 11 | | 18.6% | | 7 | | 14.0% | |  | 0.423 (1) | 0.515 | |  |
|  | Alzheimer’s | | 0 | | 0.000% | | 0 | | 0.000% | |  |  |  | |  |
|  | ETOH abuse | | 3 | | 5.08% | | 0 | | 0.000% | |  | 2.61 (1) | 0.106 | |  |
|  | Drug abuse | | 14 | | 23.7% | | 7 | | 14.0% | |  | 1.65 (1) | 0.199 | |  |

*Mean (SD) provided for continuous variables

^†^Comparison tests include independent sample t-test (for continuous variables), McNemar’s chi-squared test (for binary variables), and Extended Mantel-Haenszel (Cochran-Mantel-Haenszel) Stratified Test of Association (for categorical variables with more than 2 categories). McNemar’s chi-squared test is a special case of Cochran-Mantel-Haenszel test. Differences are statistically significant if p<0.05

^‡^Other/more than one race includes American Indian or Alaska Native, Native Hawaiian or Other Pacific Islander, Asian, and individuals with two or more races

^§^Other language includes Spanish and American Sign Language

^||^Private/commercial includes Bluecross Blueshield and Commercial

^¶^Medicaid includes Medicaid and Medicaid MCO

^#^Other payers include other, self-pay, pending Medicaid, and Tricare

**Table A.2: Patient characteristics, social risk screener responses and outcomes at baseline with complete vs. incomplete blood pressure (BP) data at six-month follow-up**

| **Outcomes/ patient characteristics** | | **Missing data** | | **Complete data** | | **Difference, mean (SE)** | **Test value**^†^**,**  **t (df)**  **or**  $\boldsymbol{x}^{\boldsymbol{2}}$ **(df)** | **p** |
| --- | --- | --- | --- | --- | --- | --- | --- | --- |
|  |  | **n** | **Mean (SD) or proportion*** | **n** | **Mean (SD) or proportion*** |  |  |  |
| **All patients** | | | | | | | | |
| Systolic BP (mmHg), mean (SD) | | 102 | 134.5 (21.5) | 296 | 132.4 (17.3) | 2.08 (2.12) | 0.982 (396) | 0.327 |
| Diastolic BP (mmHg), mean (SD) | | 102 | 82.3 (14.8) | 296 | 80.8 (12.1) | 1.47 (1.48) | 0.993 (396) | 0.321 |
|  | |  |  |  |  |  |  |  |
| *Social risk screener responses* | | |  |  |  |  |  |  |
| Within the past 12 months, we worried that our food would run out before we got money to buy more. | | 102 |  | 296 |  |  | 4.26 (3) | 0.235 |
|  | Never true | 9 | 8.82% | 20 | 6.76% |  |  |  |
|  | Sometimes true | 63 | 61.8% | 203 | 68.6% |  |  |  |
|  | Often true | 27 | 26.5% | 71 | 24.0% |  |  |  |
|  | Patient refused | 3 | 2.94% | 2 | 0.676% |  |  |  |
|  |  |  |  |  |  |  |  |  |
| Within the past 12 months, the food we bought just did not last and we did not have money to get more. | | 102 |  | 296 |  |  | 1.54 (3) | 0.674 |
|  | Never true | 14 | 13.7% | 42 | 14.2% |  |  |  |
|  | Sometimes true | 66 | 64.7% | 192 | 64.9% |  |  |  |
|  | Often true | 22 | 21.6% | 58 | 19.6% |  |  |  |
|  | Patient refused | 0 | 0.000% | 4 | 1.35% |  |  |  |
|  |  |  |  |  |  |  |  |  |
| How hard is it for you to pay for the very basics like food, housing, medical care, and heating? | | 99 |  | 292 |  |  | 2.42 (5) | 0.789 |
|  | Very hard | 14 | 14.1% | 47 | 16.1% |  |  |  |
|  | Hard | 15 | 15.2% | 57 | 19.5% |  |  |  |
|  | Somewhat hard | 49 | 49.5% | 130 | 44.5% |  |  |  |
|  | Not very hard | 14 | 14.1% | 43 | 14.7% |  |  |  |
|  | Not hard at all | 7 | 7.07% | 14 | 4.79% |  |  |  |
|  | Patient refused | 0 | 0.000% | 1 | 0.342% |  |  |  |
|  |  |  |  |  |  |  |  |  |
| Was there a time in the past 12 months when you needed to see a doctor or buy medications but could not because of cost? | | 98 |  | 291 |  |  | 1.71 (2) | 0.424 |
|  | Yes | 42 | 42.9% | 121 | 41.6% |  |  |  |
|  | No | 56 | 57.1% | 165 | 56.7% |  |  |  |
|  | Patient refused | 0 | 0.000% | 5 | 1.72% |  |  |  |
|  |  |  |  |  |  |  |  |  |
| Do you feel tense, restless, nervous, or anxious most days? | | 92 |  | 263 |  |  | 1.86 (5) | 0.869 |
|  | Very much | 16 | 17.4% | 49 | 18.6% |  |  |  |
|  | Quite a bit | 15 | 16.3% | 44 | 16.7% |  |  |  |
|  | To some extent | 19 | 20.7% | 64 | 24.3% |  |  |  |
|  | Only a little | 23 | 25.0% | 64 | 24.3% |  |  |  |
|  | Not at all | 19 | 20.7% | 41 | 15.6% |  |  |  |
|  | Patient refused | 0 | 0.000% | 1 | 0.380% |  |  |  |
|  |  |  |  |  |  |  |  |  |
| In a typical week, how many times do you talk/text with family, friends, or neighbors? | | 92 |  | 265 |  |  | 3.23 (5) | 0.664 |
|  | More than three times | 53 | 57.6% | 154 | 58.1% |  |  |  |
|  | Three times a week | 10 | 10.9% | 37 | 14.0% |  |  |  |
|  | Twice a week | 7 | 7.61% | 25 | 9.43% |  |  |  |
|  | Once a week | 15 | 16.3% | 37 | 14.0% |  |  |  |
|  | Never | 6 | 6.52% | 8 | 3.02% |  |  |  |
|  | Patient refused | 1 | 1.09% | 4 | 1.51% |  |  |  |
|  |  |  |  |  |  |  |  |  |
| How often do you get together with friends or relatives? | | 89 |  | 264 |  |  | 0.904 (5) | 0.970 |
|  | More than three times | 19 | 21.4% | 57 | 21.6% |  |  |  |
|  | Three times a week | 6 | 6.74% | 15 | 5.68% |  |  |  |
|  | Twice a week | 13 | 14.6% | 40 | 15.2% |  |  |  |
|  | Once a week | 32 | 36.0% | 104 | 39.4% |  |  |  |
|  | Never | 17 | 19.1% | 41 | 15.5% |  |  |  |
|  | Patient refused | 2 | 2.25% | 7 | 2.65% |  |  |  |
|  |  |  |  |  |  |  |  |  |
| In the last 12 months, was there a time when you did not have a steady place to sleep or slept in a shelter? | | 91 |  | 260 |  |  | **7.58 (2)** | **0.023** |
|  | Yes | 13 | 14.3% | 15 | 5.77% |  |  |  |
|  | No | 78 | 85.7% | 242 | 93.1% |  |  |  |
|  | Patient refused | 0 | 0.000% | 3 | 1.15% |  |  |  |
|  |  |  |  |  |  |  |  |  |
| Are you worried that the place you are living now is making you sick? | | 90 |  | 260 |  |  | 0.765 (2) | 0.682 |
|  | Yes | 7 | 7.78% | 21 | 8.08% |  |  |  |
|  | No | 82 | 91.1% | 232 | 89.2% |  |  |  |
|  | Patient refused | 1 | 1.11% | 7 | 2.69% |  |  |  |
|  |  |  |  |  |  |  |  |  |
| In the past 12 months, has lack of transportation kept you from medical appointments or medications? | | 100 |  | 293 |  |  | 0.345 (2) | 0.842 |
|  | Yes | 21 | 21.0% | 62 | 21.2% |  |  |  |
|  | No | 79 | 79.0% | 230 | 78.5% |  |  |  |
|  | Patient refused | 0 | 0.000% | 1 | 0.341% |  |  |  |
|  |  |  |  |  |  |  |  |  |
| *Patient characteristics* | |  |  |  |  |  |  |  |
| Age (in years), mean (SD) | | 102 | 52.0 (14.0) | 296 | 57.2 (13.3) | **-5.19 (1.55)** | **-3.36 (396)** | **0.000** |
|  |  |  |  |  |  |  |  |  |
| Gender | | 102 |  | 296 |  |  | 1.99 (1) | 0.159 |
|  | Male | 38 | 37.3% | 88 | 29.7% |  |  |  |
|  | Female | 64 | 62.8% | 208 | 70.3% |  |  |  |
|  |  |  |  |  |  |  |  |  |
| Race/ethnicity | | 102 |  | 296 |  |  | 2.28 (4) | 0.684 |
|  | Hispanic | 3 | 2.94% | 8 | 2.70% |  |  |  |
|  | Black/African American | 55 | 53.9% | 140 | 47.3% |  |  |  |
|  | Other/more than one race^‡^ | 1 | 0.980% | 8 | 2.70% |  |  |  |
|  | Patient refused/unknown | 1 | 0.980% | 2 | 0.676% |  |  |  |
|  | White | 42 | 41.2% | 138 | 46.6% |  |  |  |
|  |  |  |  |  |  |  |  |  |
| Exercise duration per week (in minutes), mean (SD) | | 89 | 9.78 (10.8) | 260 | 10.3 (11.6) | -0.486 (1.40) | -0.348 (347) | 0.728 |
|  |  |  |  |  |  |  |  |  |
| Preferred language | | 102 |  | 296 |  |  | 0.171 (1) | 0.680 |
|  | Other^§^ | 2 | 1.96% | 8 | 2.70% |  |  |  |
|  | English | 100 | 98.0% | 288 | 97.3% |  |  |  |
|  |  |  |  |  |  |  |  |  |
| Primary payer | | 102 |  | 296 |  |  | 7.13 (5) | 0.211 |
|  | Private/commercial^\|\|^ | 28 | 27.5% | 93 | 31.4% |  |  |  |
|  | Managed Care | 17 | 16.7% | 28 | 9.46% |  |  |  |
|  | Medicaid^¶^ | 17 | 16.7% | 49 | 16.6% |  |  |  |
|  | Medicare | 11 | 10.8% | 33 | 11.2% |  |  |  |
|  | Medicare Advantage | 12 | 11.8% | 56 | 18.9% |  |  |  |
|  | Others^#^ | 17 | 16.7% | 37 | 12.5% |  |  |  |
|  |  |  |  |  |  |  |  |  |
| Presence of comorbidities | | 102 |  | 296 |  |  |  |  |
|  | Cancer | 8 | 7.84% | 33 | 11.2% |  | 0.897 (1) | 0.344 |
|  | COPD | 9 | 8.82% | 41 | 13.9% |  | 1.75 (1) | 0.186 |
|  | CHF | 14 | 13.7% | 33 | 11.2% |  | 0.484 (1) | 0.487 |
|  | CVD | 16 | 15.7% | 30 | 10.1% |  | 2.29 (1) | 0.130 |
|  | Mental health disorder | 35 | 34.3% | 126 | 42.6% |  | 2.15 (1) | 0.143 |
|  | Asthma | 9 | 8.82% | 55 | 18.6% |  | **5.35 (1)** | **0.021** |
|  | Alzheimer’s | 0 | 0.000% | 1 | 0.338% |  | 0.346 (1) | 0.557 |
|  | ETOH abuse | 5 | 4.90% | 14 | 4.73% |  | 0.005 (1) | 0.944 |
|  | Drug abuse | 18 | 17.7% | 39 | 13.2% |  | 1.24 (1) | 0.266 |
|  |  |  |  |  |  |  |  |  |
|  | **Intervention group** | | | | | | | |
| Systolic BP (mmHg), mean (SD) | | 52 | 137.1 (23.2) | 147 | 133.7 (17.6) | 3.41 (3.10) | 1.10 (197) | 0.273 |
| Diastolic BP (mmHg), mean (SD) | | 52 | 83.0 (17.3) | 147 | 81.2 (13.1) | 1.81 (2.31) | 0.780 (197) | 0.436 |
|  |  |  |  |  |  |  |  |  |
| *Social risk screener responses* | | |  |  |  |  |  |  |
| Within the past 12 months, we worried that our food would run out before we got money to buy more. | | 52 |  | 147 |  |  | 1.57 (3) | 0.666 |
|  | Never true | 5 | 9.62% | 8 | 5.44% |  |  |  |
|  | Sometimes true | 32 | 61.5% | 98 | 66.7% |  |  |  |
|  | Often true | 15 | 28.9% | 40 | 27.2% |  |  |  |
|  | Patient refused | 0 | 0.000% | 1 | 0.680% |  |  |  |
|  |  |  |  |  |  |  |  |  |
| Within the past 12 months, the food we bought just did not last and we did not have money to get more. | | 52 |  | 147 |  |  | 1.79 (3) | 0.617 |
|  | Never true | 5 | 9.62% | 24 | 16.3% |  |  |  |
|  | Sometimes true | 35 | 67.3% | 91 | 61.9% |  |  |  |
|  | Often true | 12 | 23.1% | 31 | 21.1% |  |  |  |
|  | Patient refused | 0 | 0.000% | 1 | 0.680% |  |  |  |
|  |  |  |  |  |  |  |  |  |
| How hard is it for you to pay for the very basics like food, housing, medical care, and heating? | | 49 |  | 143 |  |  | 2.37 (5) | 0.797 |
|  | Very hard | 10 | 20.4% | 24 | 16.8% |  |  |  |
|  | Hard | 8 | 16.3% | 30 | 21.0% |  |  |  |
|  | Somewhat hard | 21 | 42.9% | 59 | 41.3% |  |  |  |
|  | Not very hard | 7 | 14.3% | 25 | 17.5% |  |  |  |
|  | Not hard at all | 3 | 6.12% | 4 | 2.80% |  |  |  |
|  | Patient refused | 0 | 0.000% | 1 | 0.699% |  |  |  |
|  |  |  |  |  |  |  |  |  |
| Was there a time in the past 12 months when you needed to see a doctor or buy medications but could not because of cost? | | 48 |  | 143 |  |  | 0.899 (2) | 0.638 |
|  | Yes | 24 | 50.0% | 65 | 45.5% |  |  |  |
|  | No | 24 | 50.0% | 76 | 53.2% |  |  |  |
|  | Patient refused | 0 | 0.000% | 2 | 1.40% |  |  |  |
|  |  |  |  |  |  |  |  |  |
| Do you feel tense, restless, nervous, or anxious most days? | | 49 |  | 141 |  |  | 1.27 (4) | 0.867 |
|  | Very much | 10 | 20.4% | 29 | 20.6% |  |  |  |
|  | Quite a bit | 7 | 14.3% | 28 | 19.9% |  |  |  |
|  | To some extent | 11 | 22.5% | 33 | 23.4% |  |  |  |
|  | Only a little | 12 | 24.5% | 32 | 22.7% |  |  |  |
|  | Not at all | 9 | 18.4% | 19 | 13.5% |  |  |  |
|  | Patient refused | 0 | 0.000% | 0 | 0.000% |  |  |  |
|  |  |  |  |  |  |  |  |  |
| In a typical week, how many times do you talk/text with family, friends, or neighbors? | | 49 |  | 142 |  |  | 2.43 (4) | 0.657 |
|  | More than three times | 28 | 57.1% | 78 | 54.9% |  |  |  |
|  | Three times a week | 7 | 14.3% | 21 | 14.8% |  |  |  |
|  | Twice a week | 3 | 6.12% | 14 | 9.86% |  |  |  |
|  | Once a week | 7 | 14.3% | 24 | 16.9% |  |  |  |
|  | Never | 4 | 8.16% | 5 | 3.52% |  |  |  |
|  | Patient refused | 0 | 0.000% | 0 | 0.000% |  |  |  |
|  |  |  |  |  |  |  |  |  |
| How often do you get together with friends or relatives? | | 47 |  | 142 |  |  | 5.46 (4) | 0.243 |
|  | More than three times | 10 | 21.3% | 25 | 17.6% |  |  |  |
|  | Three times a week | 5 | 10.6% | 6 | 4.23% |  |  |  |
|  | Twice a week | 5 | 10.6% | 28 | 19.7% |  |  |  |
|  | Once a week | 16 | 34.0% | 58 | 40.9% |  |  |  |
|  | Never | 11 | 23.4% | 25 | 17.6% |  |  |  |
|  | Patient refused | 0 | 0.000% | 0 | 0.000% |  |  |  |
|  |  |  |  |  |  |  |  |  |
| In the last 12 months, was there a time when you did not have a steady place to sleep or slept in a shelter? | | 49 |  | 138 |  |  | **6.12 (2)** | **0.047** |
|  | Yes | 9 | 18.4% | 9 | 6.52% |  |  |  |
|  | No | 40 | 81.6% | 128 | 92.8% |  |  |  |
|  | Patient refused | 0 | 0.000% | 1 | 0.725% |  |  |  |
|  |  |  |  |  |  |  |  |  |
| Are you worried that the place you are living now is making you sick? | | 48 |  | 138 |  |  | 0.818 (2) | 0.664 |
|  | Yes | 4 | 8.33% | 9 | 6.52% |  |  |  |
|  | No | 43 | 89.6% | 128 | 92.8% |  |  |  |
|  | Patient refused | 1 | 2.08% | 1 | 0.725% |  |  |  |
|  |  |  |  |  |  |  |  |  |
| In the past 12 months, has lack of transportation kept you from medical appointments or medications? | | 50 |  | 144 |  |  | 0.552 (2) | 0.759 |
|  | Yes | 10 | 20.0% | 33 | 22.9% |  |  |  |
|  | No | 40 | 40.0% | 110 | 76.4% |  |  |  |
|  | Patient refused | 0 | 0.000% | 1 | 0.694% |  |  |  |
|  |  |  |  |  |  |  |  |  |
| *Patient characteristics* | |  |  |  |  |  |  |  |
| Age | | 52 | 52.3 (12.5) | 147 | 57.4 (13.5) | **-5.01 (2.14)** | **-2.34 (197)** | **0.020** |
|  |  |  |  |  |  |  |  |  |
| Gender | | 52 |  | 147 |  |  | 0.028 (1) | 0.866 |
|  | Male | 18 | 34.6% | 49 | 33.3% |  |  |  |
|  | Female | 34 | 65.4% | 98 | 66.7% |  |  |  |
|  |  |  |  |  |  |  |  |  |
| Race/ethnicity | | 52 |  | 147 |  |  | 4.72 (4) | 0.317 |
|  | Hispanic | 1 | 1.92% | 5 | 3.40% |  |  |  |
|  | Black/African American | 30 | 57.7% | 62 | 42.2% |  |  |  |
|  | Other/more than one race^‡^ | 1 | 1.92% | 3 | 2.04% |  |  |  |
|  | Patient refused/unknown | 1 | 1.92% | 1 | 0.680% |  |  |  |
|  | White | 19 | 36.5% | 76 | 51.7% |  |  |  |
|  |  |  |  |  |  |  |  |  |
| Exercise duration per week (in minutes), mean (SD) | | 48 | 9.69 (10.6) | 140 | 9.91 (11.8) | -0.227 (1.92) | -0.118 (186) | 0.906 |
|  |  |  |  |  |  |  |  |  |
| Preferred language | | 52 |  | 147 |  |  | 0.205 (1) | 0.651 |
|  | Other^§^ | 2 | 3.85% | 8 | 5.44% |  |  |  |
|  | English | 50 | 96.2% | 139 | 94.6% |  |  |  |
|  |  |  |  |  |  |  |  |  |
| Primary payer | | 52 |  | 147 |  |  | 1.00 (5) | 0.963 |
|  | Private/commercial^\|\|^ | 14 | 26.9% | 42 | 28.6% |  |  |  |
|  | Managed Care | 7 | 13.5% | 13 | 8.84% |  |  |  |
|  | Medicaid^¶^ | 10 | 19.2% | 28 | 19.1% |  |  |  |
|  | Medicare | 6 | 11.5% | 20 | 13.6% |  |  |  |
|  | Medicare Advantage | 8 | 15.4% | 24 | 16.3% |  |  |  |
|  | Others^#^ | 7 | 13.5% | 20 | 13.6% |  |  |  |
|  |  |  |  |  |  |  |  |  |
| Presence of comorbidities | | 52 |  | 147 |  |  |  |  |
|  | Cancer | 2 | 3.85% | 17 | 11.6% |  | 2.65 (1) | 0.104 |
|  | COPD | 4 | 7.69% | 19 | 12.9% |  | 1.03 (1) | 0.310 |
|  | CHF | 8 | 15.4% | 17 | 11.6% |  | 0.510 (1) | 0.475 |
|  | CVD | 11 | 21.2% | 17 | 11.6% |  | 2.92 (1) | 0.087 |
|  | Mental health disorder | 18 | 34.6% | 58 | 39.5% |  | 0.381 (1) | 0.537 |
|  | Asthma | 4 | 7.69% | 24 | 16.3% |  | 2.37 (1) | 0.124 |
|  | Alzheimer’s | 0 | 0.000% | 1 | 0.680% |  | 0.356 (1) | 0.551 |
|  | ETOH abuse | 3 | 5.77% | 10 | 6.80% |  | 0.067 (1) | 0.795 |
|  | Drug abuse | 7 | 13.5% | 13 | 8.84% |  | 0.906 (1) | 0.341 |
|  |  |  |  |  |  |  |  |  |
|  | **Control group** | | | | | | | |
| Systolic BP (mmHg), mean (SD) | | 50 | 131.7 (19.4) | 149 | 131.1 (16.9) | 0.626 (2.87) | 0.218 (197) | 0.828 |
| Diastolic BP (mmHg), mean (SD) | | 50 | 81.6 (11.9) | 149 | 80.5 (11.1) | 1.10 (1.85) | 0.597 (197) | 0.551 |
|  |  |  |  |  |  |  |  |  |
| *Social risk screener responses* | | |  |  |  |  |  |  |
| Within the past 12 months, we worried that our food would run out before we got money to buy more. | | 50 |  | 149 |  |  | 5.86 (3) | 0.119 |
|  | Never true | 4 | 8.00% | 12 | 8.05% |  |  |  |
|  | Sometimes true | 31 | 62.0% | 105 | 70.5% |  |  |  |
|  | Often true | 12 | 24.0% | 31 | 20.8% |  |  |  |
|  | Patient refused | 3 | 6.00% | 1 | 0.671% |  |  |  |
|  |  |  |  |  |  |  |  |  |
| Within the past 12 months, the food we bought just did not last and we did not have money to get more. | | 50 |  | 149 |  |  | 2.23 (3) | 0.525 |
|  | Never true | 9 | 18.0% | 18 | 12.1% |  |  |  |
|  | Sometimes true | 31 | 62.0% | 101 | 67.8% |  |  |  |
|  | Often true | 10 | 20.0% | 27 | 18.1% |  |  |  |
|  | Patient refused | 0 | 0.000% | 3 | 2.01% |  |  |  |
|  |  |  |  |  |  |  |  |  |
| How hard is it for you to pay for the very basics like food, housing, medical care, and heating? | | 50 |  | 149 |  |  | 2.62 (4) | 0.623 |
|  | Very hard | 4 | 8.00% | 23 | 15.4% |  |  |  |
|  | Hard | 7 | 14.0% | 27 | 18.1% |  |  |  |
|  | Somewhat hard | 28 | 56.0% | 71 | 47.7% |  |  |  |
|  | Not very hard | 7 | 14.0% | 18 | 12.1% |  |  |  |
|  | Not hard at all | 4 | 8.00% | 10 | 6.71% |  |  |  |
|  | Patient refused | 0 | 0.000% | 0 | 0.000% |  |  |  |
|  |  |  |  |  |  |  |  |  |
| Was there a time in the past 12 months when you needed to see a doctor or buy medications but could not because of cost? | | 50 |  | 148 |  |  | 1.14 (2) | 0.566 |
|  | Yes | 18 | 36.0% | 56 | 37.8% |  |  |  |
|  | No | 32 | 64.0% | 89 | 60.1% |  |  |  |
|  | Patient refused | 0 | 0.000% | 3 | 2.03% |  |  |  |
|  |  |  |  |  |  |  |  |  |
| Do you feel tense, restless, nervous, or anxious most days? | | 43 |  | 122 |  |  | 2.21 (5) | 0.820 |
|  | Very much | 6 | 14.0% | 20 | 16.4% |  |  |  |
|  | Quite a bit | 8 | 18.6% | 16 | 13.1% |  |  |  |
|  | To some extent | 8 | 18.6% | 31 | 25.4% |  |  |  |
|  | Only a little | 11 | 25.6% | 32 | 26.2% |  |  |  |
|  | Not at all | 10 | 23.3% | 22 | 18.0% |  |  |  |
|  | Patient refused | 0 | 0.000% | 1 | 0.820% |  |  |  |
|  |  |  |  |  |  |  |  |  |
| In a typical week, how many times do you talk/text with family, friends, or neighbors? | | 43 |  | 123 |  |  | 3.32 (5) | 0.651 |
|  | More than three times | 25 | 58.1% | 76 | 61.8% |  |  |  |
|  | Three times a week | 3 | 6.98% | 16 | 13.0% |  |  |  |
|  | Twice a week | 4 | 9.30% | 11 | 8.94% |  |  |  |
|  | Once a week | 8 | 18.6% | 13 | 10.6% |  |  |  |
|  | Never | 2 | 4.65% | 3 | 2.44% |  |  |  |
|  | Patient refused | 1 | 2.33% | 4 | 3.25% |  |  |  |
|  |  |  |  |  |  |  |  |  |
| How often do you get together with friends or relatives? | | 42 |  | 122 |  |  | 3.83 (5) | 0.574 |
|  | More than three times | 9 | 21.4% | 32 | 26.2% |  |  |  |
|  | Three times a week | 1 | 2.38% | 9 | 7.38% |  |  |  |
|  | Twice a week | 8 | 19.1% | 12 | 9.84% |  |  |  |
|  | Once a week | 16 | 38.1% | 46 | 37.7% |  |  |  |
|  | Never | 6 | 14.3% | 16 | 13.1% |  |  |  |
|  | Patient refused | 2 | 4.76% | 7 | 5.74% |  |  |  |
|  |  |  |  |  |  |  |  |  |
| In the last 12 months, was there a time when you did not have a steady place to sleep or slept in a shelter? | | 42 |  | 122 |  |  | 1.81 (2) | 0.406 |
|  | Yes | 4 | 9.52% | 6 | 4.92% |  |  |  |
|  | No | 38 | 90.5% | 114 | 93.4% |  |  |  |
|  | Patient refused | 0 | 0.000% | 2 | 1.64% |  |  |  |
|  |  |  |  |  |  |  |  |  |
| Are you worried that the place you are living now is making you sick? | | 42 |  | 122 |  |  | 2.52 (2) | 0.284 |
|  | Yes | 3 | 7.14% | 12 | 9.84% |  |  |  |
|  | No | 39 | 92.9% | 104 | 85.3% |  |  |  |
|  | Patient refused | 0 | 0.000% | 6 | 4.92% |  |  |  |
|  |  |  |  |  |  |  |  |  |
| In the past 12 months, has lack of transportation kept you from medical appointments or medications? | | 50 |  | 149 |  |  | 0.150 (1) | 0.699 |
|  | Yes | 11 | 22.0% | 29 | 19.5% |  |  |  |
|  | No | 39 | 78.0% | 120 | 80.5% |  |  |  |
|  | Patient refused | 0 | 0.000% | 0 | 0.000% |  |  |  |
|  |  |  |  |  |  |  |  |  |
| *Patient characteristics* | |  |  |  |  |  |  |  |
| Age (in years), mean (SD) | | 50 | 51.7 (15.4) | 149 | 57.1 (13.1) | **-5.39 (2.24)** | **-2.40 (197)** | **0.017** |
|  |  |  |  |  |  |  |  |  |
| Gender | | 50 |  | 149 |  |  | 3.43 (1) | 0.064 |
|  | Male | 20 | 40.0% | 39 | 26.2% |  |  |  |
|  | Female | 30 | 60.0% | 110 | 73.8% |  |  |  |
|  |  |  |  |  |  |  |  |  |
| Race/ethnicity | | 50 |  | 149 |  |  | 2.81 (4) | 0.590 |
|  | Hispanic | 2 | 4.00% | 3 | 2.01% |  |  |  |
|  | Black/African American | 25 | 50.0% | 78 | 52.4% |  |  |  |
|  | Other/more than one race^‡^ | 0 | 0.000% | 5 | 3.36% |  |  |  |
|  | Patient refused/unknown | 0 | 0.000% | 1 | 0.671% |  |  |  |
|  | White | 23 | 46.0% | 62 | 41.6% |  |  |  |
|  |  |  |  |  |  |  |  |  |
| Exercise duration per week (in minutes), mean (SD) | | 41 | 9.88 (11.1) | 120 | 10.7 (11.4) | -0.789 (2.05) | -0.385 (159) | 0.701 |
|  |  |  |  |  |  |  |  |  |
| Preferred language | | 50 |  | 149 |  |  |  |  |
|  | Other^§^ | 0 | 0.000% | 0 | 0.000% |  |  |  |
|  | English | 50 | 100% | 149 | 100% |  |  |  |
|  |  |  |  |  |  |  |  |  |
| Primary payer | | 50 |  | 149 |  |  | 9.25 (5) | 0.100 |
|  | Private/commercial^\|\|^ | 14 | 28.0% | 51 | 34.2% |  |  |  |
|  | Managed Care | 10 | 20.0% | 15 | 10.1% |  |  |  |
|  | Medicaid^¶^ | 7 | 14.0% | 21 | 14.1% |  |  |  |
|  | Medicare | 5 | 10.0% | 13 | 8.72% |  |  |  |
|  | Medicare Advantage | 4 | 8.00% | 32 | 21.5% |  |  |  |
|  | Others^#^ | 10 | 20.0% | 17 | 11.4% |  |  |  |
|  |  |  |  |  |  |  |  |  |
| Presence of comorbidities | | 50 |  | 149 |  |  |  |  |
|  | Cancer | 6 | 12.0% | 16 | 10.7% |  | 0.061 (1) | 0.806 |
|  | COPD | 5 | 10.0% | 22 | 14.8% |  | 0.725 (1) | 0.395 |
|  | CHF | 6 | 12.0% | 16 | 10.7% |  | 0.061 (1) | 0.806 |
|  | CVD | 5 | 10.0% | 13 | 8.72% |  | 0.074 (1) | 0.786 |
|  | Mental health disorder | 17 | 34.0% | 68 | 45.6% |  | 2.07 (1) | 0.150 |
|  | Asthma | 5 | 10.0% | 31 | 20.8% |  | 2.95 (1) | 0.086 |
|  | Alzheimer’s | 0 | 0.000% | 0 | 0.000% |  |  |  |
|  | ETOH abuse | 2 | 4.00% | 4 | 2.68% |  | 0.222 (1) | 0.638 |
|  | Drug abuse | 11 | 22.0% | 26 | 17.5% |  | 0.512 (1) | 0.474 |

*Mean (SD) provided for continuous variables

^†^Comparison tests include independent sample t-test (for continuous variables), McNemar’s chi-squared test (for binary variables), and Extended Mantel-Haenszel (Cochran-Mantel-Haenszel) Stratified Test of Association (for categorical variables with more than 2 categories). McNemar’s chi-squared test is a special case of Cochran-Mantel-Haenszel test. Differences are statistically significant if p<0.05

^‡^Other/more than one race includes American Indian or Alaska Native, Native Hawaiian or Other Pacific Islander, Asian, and individuals with two or more races

^§^Other language includes Spanish and American Sign Language

^||^Private/commercial includes Bluecross Blueshield and Commercial

^¶^Medicaid includes Medicaid and Medicaid MCO

^#^Other payers include other, self-pay, pending Medicaid, and Tricare

**Table A.3: Patient characteristics, social risk screener responses and outcomes at baseline with complete vs. missing BMI data at six-month follow-up**

| **Outcomes/ patient characteristics** | | **Missing data** | | **Complete data** | | **Difference, mean (SE)** | **Test value**^†^**,**  **t (df)**  **or**  $\boldsymbol{x}^{\boldsymbol{2}}$ **(df)** | **p** |
| --- | --- | --- | --- | --- | --- | --- | --- | --- |
|  |  | **n** | **Mean (SD) or proportion*** | **n** | **Mean (SD) or proportion*** |  |  |  |
| **All patients** | | | | | | | | |
| BMI, mean (SD) | | 117 | 35.6 (11.8) | 319 | 35.4 (9.42) | 0.148 (1.09) | 0.135 (434) | 0.893 |
|  | |  |  |  |  |  |  |  |
| *Social risk screener responses* | | |  |  |  |  |  |  |
| Within the past 12 months, we worried that our food would run out before we got money to buy more. | | 119 |  | 319 |  |  | 4.22 (3) | 0.239 |
|  | Never true | 11 | 9.24% | 20 | 6.27% |  |  |  |
|  | Sometimes true | 74 | 62.2% | 216 | 67.8% |  |  |  |
|  | Often true | 31 | 26.1% | 81 | 25.4% |  |  |  |
|  | Patient refused | 3 | 2.52% | 2 | 0.627% |  |  |  |
|  |  |  |  |  |  |  |  |  |
| Within the past 12 months, the food we bought just did not last and we did not have money to get more. | | 119 |  | 319 |  |  | 1.82 (3) | 0.610 |
|  | Never true | 15 | 12.6% | 46 | 14.4% |  |  |  |
|  | Sometimes true | 79 | 66.4% | 202 | 63.3% |  |  |  |
|  | Often true | 25 | 21.0% | 67 | 21.0% |  |  |  |
|  | Patient refused | 0 | 0.000% | 4 | 1.25% |  |  |  |
|  |  |  |  |  |  |  |  |  |
| How hard is it for you to pay for the very basics like food, housing, medical care, and heating? | | 116 |  | 314 |  |  | 2.83 (5) | 0.726 |
|  | Very hard | 18 | 15.5% | 53 | 16.9% |  |  |  |
|  | Hard | 17 | 14.7% | 62 | 19.8% |  |  |  |
|  | Somewhat hard | 56 | 48.3% | 136 | 43.3% |  |  |  |
|  | Not very hard | 16 | 13.8% | 46 | 14.7% |  |  |  |
|  | Not hard at all | 8 | 6.90% | 16 | 5.10% |  |  |  |
|  | Patient refused | 1 | 0.862% | 1 | 0.318% |  |  |  |
|  |  |  |  |  |  |  |  |  |
| Was there a time in the past 12 months when you needed to see a doctor or buy medications but could not because of cost? | | 115 |  | 313 |  |  | 1.19 (2) | 0.552 |
|  | Yes | 55 | 47.8% | 133 | 42.5% |  |  |  |
|  | No | 59 | 51.3% | 175 | 55.9% |  |  |  |
|  | Patient refused | 1 | 0.870% | 5 | 1.60% |  |  |  |
|  |  |  |  |  |  |  |  |  |
| Do you feel tense, restless, nervous, or anxious most days? | | 109 |  | 284 |  |  | 0.804 (5) | 0.977 |
|  | Very much | 20 | 18.4% | 54 | 19.0% |  |  |  |
|  | Quite a bit | 18 | 16.5% | 50 | 17.6% |  |  |  |
|  | To some extent | 24 | 22.0% | 66 | 23.2% |  |  |  |
|  | Only a little | 28 | 25.7% | 70 | 24.7% |  |  |  |
|  | Not at all | 19 | 17.4% | 43 | 15.1% |  |  |  |
|  | Patient refused | 0 | 0.000% | 1 | 0.352% |  |  |  |
|  |  |  |  |  |  |  |  |  |
| In a typical week, how many times do you talk/text with family, friends, or neighbors? | | 109 |  | 286 |  |  | 1.95 (5) | 0.856 |
|  | More than three times | 64 | 58.7% | 165 | 57.7% |  |  |  |
|  | Three times a week | 11 | 10.1% | 38 | 13.3% |  |  |  |
|  | Twice a week | 10 | 9.17% | 28 | 9.79% |  |  |  |
|  | Once a week | 16 | 14.7% | 42 | 14.7% |  |  |  |
|  | Never | 6 | 5.50% | 9 | 3.15% |  |  |  |
|  | Patient refused | 2 | 1.83% | 4 | 1.40% |  |  |  |
|  |  |  |  |  |  |  |  |  |
| How often do you get together with friends or relatives? | | 106 |  | 285 |  |  | 0.542 (5) | 0.991 |
|  | More than three times | 22 | 20.8% | 61 | 21.4% |  |  |  |
|  | Three times a week | 8 | 7.55% | 18 | 6.32% |  |  |  |
|  | Twice a week | 17 | 16.0% | 43 | 15.1% |  |  |  |
|  | Once a week | 38 | 35.9% | 111 | 39.0% |  |  |  |
|  | Never | 18 | 17.0% | 45 | 15.8% |  |  |  |
|  | Patient refused | 3 | 2.83% | 7 | 2.46% |  |  |  |
|  |  |  |  |  |  |  |  |  |
| In the last 12 months, was there a time when you did not have a steady place to sleep or slept in a shelter? | | 108 |  | 280 |  |  | **12.7 (2)** | **0.002** |
|  | Yes | 18 | 16.7% | 16 | 5.71% |  |  |  |
|  | No | 90 | 83.3% | 261 | 93.2% |  |  |  |
|  | Patient refused | 0 | 0.000% | 3 | 1.07% |  |  |  |
|  |  |  |  |  |  |  |  |  |
| Are you worried that the place you are living now is making you sick? | | 107 |  | 281 |  |  | 0.706 (2) | 0.703 |
|  | Yes | 11 | 10.3% | 22 | 7.83% |  |  |  |
|  | No | 94 | 87.9% | 252 | 89.7% |  |  |  |
|  | Patient refused | 2 | 1.87% | 7 | 2.49% |  |  |  |
|  |  |  |  |  |  |  |  |  |
| In the past 12 months, has lack of transportation kept you from medical appointments or medications? | | 117 |  | 315 |  |  | 0.683 (2) | 0.711 |
|  | Yes | 26 | 22.2% | 65 | 20.6% |  |  |  |
|  | No | 90 | 76.9% | 249 | 79.1% |  |  |  |
|  | Patient refused | 1 | 0.855% | 1 | 0.317% |  |  |  |
|  |  |  |  |  |  |  |  |  |
| *Patient characteristics* | |  |  |  |  |  |  |  |
| Age (in years), mean (SD) | | 119 | 50.8 (14.1) | 319 | 56.2 (13.8) | **-5.34 (1.49)** | **-3.59 (436)** | **0.000** |
|  |  |  |  |  |  |  |  |  |
| Gender | | 119 |  | 219 |  |  | 1.31 (1) | 0.253 |
|  | Male | 43 | 36.1% | 97 | 30.4% |  |  |  |
|  | Female | 76 | 63.9% | 222 | 69.6% |  |  |  |
|  |  |  |  |  |  |  |  |  |
| Race/ethnicity | | 119 |  | 319 |  |  | 2.61 (4) | 0.625 |
|  | Hispanic | 5 | 4.20% | 16 | 5.02% |  |  |  |
|  | Black/African American | 63 | 52.9% | 147 | 46.1% |  |  |  |
|  | Other/more than one race^‡^ | 1 | 0.840% | 8 | 2.51% |  |  |  |
|  | Patient refused/unknown | 1 | 0.840% | 2 | 0.627% |  |  |  |
|  | White | 49 | 41.2% | 146 | 45.8% |  |  |  |
|  |  |  |  |  |  |  |  |  |
| Exercise duration per week (in minutes), mean (SD) | | 106 | 10.6 (11.2) | 281 | 10.2 (11.6) | 0. 375 (1.31) | 0.285 (385) | 0.776 |
|  |  |  |  |  |  |  |  |  |
| Preferred language | | 119 |  | 319 |  |  | 1.50 (1) | 0.220 |
|  | Other^§^ | 2 | 1.68% | 13 | 4.08% |  |  |  |
|  | English | 117 | 98.3% | 306 | 95.9% |  |  |  |
|  |  |  |  |  |  |  |  |  |
| Primary payer | | 118 |  | 319 |  |  | 6.81 (5) | 0.236 |
|  | Private/commercial^\|\|^ | 33 | 28.0% | 95 | 29.8% |  |  |  |
|  | Managed Care | 22 | 18.6% | 37 | 11.6% |  |  |  |
|  | Medicaid^¶^ | 21 | 17.8% | 55 | 17.2% |  |  |  |
|  | Medicare | 11 | 9.32% | 33 | 10.3% |  |  |  |
|  | Medicare Advantage | 12 | 10.2% | 56 | 17.6% |  |  |  |
|  | Others^#^ | 19 | 16.1% | 43 | 13.5% |  |  |  |
|  |  |  |  |  |  |  |  |  |
| Presence of comorbidities | | 119 |  | 319 |  |  |  |  |
|  | Cancer | 9 | 7.56% | 34 | 10.7% |  | 0.938 (1) | 0.333 |
|  | COPD | 9 | 7.56% | 42 | 13.2% |  | 2.64 (1) | 0.104 |
|  | CHF | 15 | 12.6% | 33 | 10.3% |  | 0.454 (1) | 0.501 |
|  | CVD | 16 | 13.5% | 30 | 9.40% |  | 1.51 (1) | 0.220 |
|  | Mental health disorder | 41 | 34.5% | 132 | 41.4% |  | 1.74 (1) | 0.187 |
|  | Asthma | 11 | 9.24% | 55 | 17.2% |  | **4.33 (1)** | **0.037** |
|  | Alzheimer’s | 0 | 0.000% | 1 | 0.314% |  | 0.374 (1) | 0.541 |
|  | ETOH abuse | 8 | 6.72% | 16 | 5.02% |  | 0.488 (1) | 0.485 |
|  | Drug abuse |  |  |  |  |  |  |  |
|  |  |  |  |  |  |  |  |  |
|  | **Intervention group** | | | | | | | |
| BMI, mean (SD) | | 56 | 36.5 (11.8) | 161 | 34.6 (8.80) | 1.89 (1.50) | 1.26 (215) | 0.209 |
|  |  |  |  |  |  |  |  |  |
| *Social risk screener responses* | | |  |  |  |  |  |  |
| Within the past 12 months, we worried that our food would run out before we got money to buy more. | | 58 |  | 161 |  |  | 1.89 (3) | 0.595 |
|  | Never true | 6 | 10.3% | 9 | 5.59% |  |  |  |
|  | Sometimes true | 37 | 63.8% | 105 | 65.2% |  |  |  |
|  | Often true | 15 | 25.9% | 46 | 28.6% |  |  |  |
|  | Patient refused | 0 | 0.000% | 1 | 0.621% |  |  |  |
|  |  |  |  |  |  |  |  |  |
| Within the past 12 months, the food we bought just did not last and we did not have money to get more. | | 58 |  | 161 |  |  | 1.85 (3) | 0.605 |
|  | Never true | 6 | 10.3% | 25 | 15.5% |  |  |  |
|  | Sometimes true | 40 | 69.0% | 97 | 60.3% |  |  |  |
|  | Often true | 12 | 20.7% | 38 | 23.6% |  |  |  |
|  | Patient refused | 0 | 0.000% | 1 | 0.621% |  |  |  |
|  |  |  |  |  |  |  |  |  |
| How hard is it for you to pay for the very basics like food, housing, medical care, and heating? | | 55 |  | 157 |  |  | 3.29 (5) | 0.655 |
|  | Very hard | 11 | 20.0% | 29 | 18.5% |  |  |  |
|  | Hard | 8 | 14.6% | 32 | 20.4% |  |  |  |
|  | Somewhat hard | 24 | 43.6% | 61 | 38.9% |  |  |  |
|  | Not very hard | 7 | 12.7% | 28 | 17.8% |  |  |  |
|  | Not hard at all | 4 | 7.27% | 6 | 3.82% |  |  |  |
|  | Patient refused | 1 | 1.82% | 1 | 0.637% |  |  |  |
|  |  |  |  |  |  |  |  |  |
| Was there a time in the past 12 months when you needed to see a doctor or buy medications but could not because of cost? | | 54 |  | 157 |  |  | 0.614 (2) | 0.736 |
|  | Yes | 28 | 51.9% | 73 | 46.5% |  |  |  |
|  | No | 25 | 46.3% | 82 | 52.2% |  |  |  |
|  | Patient refused | 1 | 1.85% | 2 | 1.27% |  |  |  |
|  |  |  |  |  |  |  |  |  |
| Do you feel tense, restless, nervous, or anxious most days? | | 55 |  | 154 |  |  | 0.857 (4) | 0.931 |
|  | Very much | 11 | 20.0% | 31 | 20.1% |  |  |  |
|  | Quite a bit | 9 | 16.4% | 32 | 20.8% |  |  |  |
|  | To some extent | 12 | 21.8% | 36 | 23.4% |  |  |  |
|  | Only a little | 14 | 25.5% | 34 | 22.1% |  |  |  |
|  | Not at all | 9 | 16.4% | 21 | 13.6% |  |  |  |
|  | Patient refused | 0 | 0.000% | 0 | 0.000% |  |  |  |
|  |  |  |  |  |  |  |  |  |
| In a typical week, how many times do you talk/text with family, friends, or neighbors? | | 55 |  | 155 |  |  | 5.57 (5) | 0.350 |
|  | More than three times | 32 | 58.2% | 83 | 53.6% |  |  |  |
|  | Three times a week | 7 | 12.7% | 21 | 13.6% |  |  |  |
|  | Twice a week | 3 | 5.45% | 17 | 11.0% |  |  |  |
|  | Once a week | 8 | 14.6% | 28 | 18.1% |  |  |  |
|  | Never | 4 | 7.27% | 6 | 3.87% |  |  |  |
|  | Patient refused | 1 | 1.82% | 0 | 0.000% |  |  |  |
|  |  |  |  |  |  |  |  |  |
| How often do you get together with friends or relatives? | | 53 |  | 155 |  |  | 7.23 (5) | 0.204 |
|  | More than three times | 11 | 20.8% | 28 | 18.1% |  |  |  |
|  | Three times a week | 5 | 9.43% | 7 | 4.52% |  |  |  |
|  | Twice a week | 6 | 11.3% | 30 | 19.4% |  |  |  |
|  | Once a week | 18 | 34.0% | 63 | 40.7% |  |  |  |
|  | Never | 12 | 22.6% | 27 | 17.4% |  |  |  |
|  | Patient refused | 1 | 1.89% | 0 | 0.000% |  |  |  |
|  |  |  |  |  |  |  |  |  |
| In the last 12 months, was there a time when you did not have a steady place to sleep or slept in a shelter? | | 55 |  | 151 |  |  | 5.53 (2) | 0.063 |
|  | Yes | 10 | 18.2% | 11 | 7.28% |  |  |  |
|  | No | 45 | 81.8% | 139 | 92.1% |  |  |  |
|  | Patient refused | 0 | 0.000% | 1 | 0.662% |  |  |  |
|  |  |  |  |  |  |  |  |  |
| Are you worried that the place you are living now is making you sick? | | 54 |  | 151 |  |  | 3.03 (2) | 0.220 |
|  | Yes | 5 | 9.26% | 10 | 6.62% |  |  |  |
|  | No | 47 | 87.0% | 140 | 92.7% |  |  |  |
|  | Patient refused | 2 | 3.70% | 1 | 0.662% |  |  |  |
|  |  |  |  |  |  |  |  |  |
| In the past 12 months, has lack of transportation kept you from medical appointments or medications? | | 56 |  | 158 |  |  | 0.599 (2) | 0.741 |
|  | Yes | 12 | 21.4% | 35 | 22.2% |  |  |  |
|  | No | 43 | 76.8% | 122 | 77.2% |  |  |  |
|  | Patient refused | 1 | 1.79% | 1 | 0.633% |  |  |  |
|  |  |  |  |  |  |  |  |  |
| *Patient characteristics* | |  |  |  |  |  |  |  |
| Age | | 58 | 52.3 (12.3) | 161 | 56.0 (14.1) | -3.74 (2.10) | -1.78 (217) | 0.076 |
|  |  |  |  |  |  |  |  |  |
| Gender | | 58 |  | 161 |  | 0.078 (1) | 0.781 |  |
|  | Male | 19 | 32.8% | 56 | 34.8% |  |  |  |
|  | Female | 39 | 67.2% | 105 | 65.2% |  |  |  |
|  |  |  |  |  |  |  |  |  |
| Race/ethnicity | | 58 |  | 161 |  | 6.40 (4) | 0.171 |  |
|  | Hispanic | 1 | 1.72% | 10 | 6.21% |  |  |  |
|  | Black/African American | 33 | 56.9% | 65 | 40.4% |  |  |  |
|  | Other/more than one race^‡^ | 1 | 1.72% | 3 | 1.86% |  |  |  |
|  | Patient refused/unknown | 1 | 1.72% | 1 | 0.621% |  |  |  |
|  | White | 22 | 37.9% | 82 | 50.9% |  |  |  |
|  |  |  |  |  |  |  |  |  |
| Exercise duration per week (in minutes), mean (SD) | | 54 | 9.69 (10.8) | 153 | 9.71 (11.7) | -0.027 (1.82) | -0.015 (205) | 0.988 |
|  |  |  |  |  |  |  |  |  |
| Preferred language | | 58 |  | 161 |  |  | 1.14 (1) | 0.285 |
|  | Other^§^ | 2 | 3.45% | 12 | 7.45% |  |  |  |
|  | English | 56 | 96.6% | 149 | 92.6% |  |  |  |
|  |  |  |  |  |  |  |  |  |
| Primary payer | | 57 |  | 161 |  |  | 1.72 (5) | 0.886 |
|  | Private/commercial^\|\|^ | 16 | 28.1% | 44 | 27.3% |  |  |  |
|  | Managed Care | 9 | 15.8% | 16 | 9.94% |  |  |  |
|  | Medicaid^¶^ | 11 | 19.3% | 32 | 19.9% |  |  |  |
|  | Medicare | 6 | 10.5% | 20 | 12.4% |  |  |  |
|  | Medicare Advantage | 8 | 14.0% | 24 | 14.9% |  |  |  |
|  | Others^#^ | 7 | 12.3% | 25 | 15.5% |  |  |  |
|  |  |  |  |  |  |  |  |  |
| Presence of comorbidities | | 58 |  | 161 |  |  |  |  |
|  | Cancer | 2 | 3.45% | 18 | 11.2% |  | 3.07 (1) | 0.080 |
|  | COPD | 4 | 6.90% | 20 | 12.4% |  | 1.33 (1) | 0.248 |
|  | CHF | 9 | 15.5% | 17 | 10.6% |  | 1.00 (1) | 0.317 |
|  | CVD | 11 | 19.0% | 17 | 10.6% |  | 2.70 (1) | 0.100 |
|  | Mental health disorder | 20 | 34.5% | 62 | 38.5% |  | 0.295 (1) | 0.587 |
|  | Asthma | 4 | 6.90% | 24 | 14.9% |  | 2.45 (1) | 0.117 |
|  | Alzheimer’s | 0 | 0.000% | 1 | 0.621% |  | 0.362 (1) | 0.547 |
|  | ETOH abuse | 3 | 5.17% | 12 | 7.45% |  | 0.348 (1) | 0.555 |
|  | Drug abuse | 8 | 13.8% | 17 | 10.6% |  | 0.441 (1) | 0.507 |
|  |  |  |  |  |  |  |  |  |
|  | **Control group** | | | | | | | |
| BMI, mean (SD) | | 61 | 34.8 (11.8) | 158 | 36.3 (9.96) | -1.54 (1.58) | -0.973 (217) | 0.332 |
|  |  |  |  |  |  |  |  |  |
| *Social risk screener responses* | | |  |  |  |  |  |  |
| Within the past 12 months, we worried that our food would run out before we got money to buy more. | | 61 |  | 158 |  |  | 5.43 (3) | 0.143 |
|  | Never true | 5 | 8.20% | 11 | 6.96% |  |  |  |
|  | Sometimes true | 37 | 60.7% | 111 | 70.3% |  |  |  |
|  | Often true | 16 | 26.2% | 35 | 22.2% |  |  |  |
|  | Patient refused | 3 | 4.92% | 1 | 0.633% |  |  |  |
|  |  |  |  |  |  |  |  |  |
| Within the past 12 months, the food we bought just did not last and we did not have money to get more. | | 61 |  | 158 |  |  | 1.47 (3) | 0.689 |
|  | Never true | 9 | 14.8% | 21 | 13.3% |  |  |  |
|  | Sometimes true | 39 | 63.9% | 105 | 66.6% |  |  |  |
|  | Often true | 13 | 21.3% | 29 | 18.4% |  |  |  |
|  | Patient refused | 0 | 0.000% | 3 | 1.90% |  |  |  |
|  |  |  |  |  |  |  |  |  |
| How hard is it for you to pay for the very basics like food, housing, medical care, and heating? | | 61 |  | 157 |  |  | 1.50 (4) | 0.827 |
|  | Very hard | 7 | 11.5% | 24 | 15.3% |  |  |  |
|  | Hard | 9 | 14.8% | 30 | 19.1% |  |  |  |
|  | Somewhat hard | 32 | 52.5% | 75 | 47.8% |  |  |  |
|  | Not very hard | 9 | 14.8% | 18 | 11.5% |  |  |  |
|  | Not hard at all | 4 | 6.56% | 10 | 6.37% |  |  |  |
|  | Patient refused | 0 | 0.000% | 0 | 0.000% |  |  |  |
|  |  |  |  |  |  |  |  |  |
| Was there a time in the past 12 months when you needed to see a doctor or buy medications but could not because of cost? | | 61 |  | 156 |  |  | 1.65 (2) | 0.437 |
|  | Yes | 27 | 44.3% | 60 | 38.5% |  |  |  |
|  | No | 34 | 55.7% | 93 | 59.6% |  |  |  |
|  | Patient refused | 0 | 0.000% | 3 | 1.92% |  |  |  |
|  |  |  |  |  |  |  |  |  |
| Do you feel tense, restless, nervous, or anxious most days? | | 54 |  | 130 |  |  | 0.757 (5) | 0.980 |
|  | Very much | 9 | 16.7% | 23 | 17.7% |  |  |  |
|  | Quite a bit | 9 | 16.7% | 18 | 13.8% |  |  |  |
|  | To some extent | 12 | 22.2% | 30 | 23.1% |  |  |  |
|  | Only a little | 14 | 25.9% | 36 | 27.7% |  |  |  |
|  | Not at all | 10 | 18.5% | 22 | 16.9% |  |  |  |
|  | Patient refused | 0 | 0.000% | 1 | 0.769% |  |  |  |
|  |  |  |  |  |  |  |  |  |
| In a typical week, how many times do you talk/text with family, friends, or neighbors? | | 54 |  | 131 |  |  | 2.97 (5) | 0.705 |
|  | More than three times | 32 | 59.3% | 82 | 62.6% |  |  |  |
|  | Three times a week | 4 | 7.41% | 17 | 13.0% |  |  |  |
|  | Twice a week | 7 | 13.0% | 11 | 8.40% |  |  |  |
|  | Once a week | 8 | 14.8% | 14 | 10.7% |  |  |  |
|  | Never | 2 | 3.70% | 3 | 2.29% |  |  |  |
|  | Patient refused | 1 | 1.85% | 4 | 3.05% |  |  |  |
|  |  |  |  |  |  |  |  |  |
| How often do you get together with friends or relatives? | | 53 |  | 130 |  |  | 4.43 (5) | 0.489 |
|  | More than three times | 11 | 20.8% | 33 | 25.4% |  |  |  |
|  | Three times a week | 3 | 5.66% | 11 | 8.46% |  |  |  |
|  | Twice a week | 11 | 20.8% | 13 | 10.0% |  |  |  |
|  | Once a week | 20 | 37.7% | 48 | 36.9% |  |  |  |
|  | Never | 6 | 11.3% | 18 | 13.8% |  |  |  |
|  | Patient refused | 2 | 3.77% | 7 | 5.39% |  |  |  |
|  |  |  |  |  |  |  |  |  |
| In the last 12 months, was there a time when you did not have a steady place to sleep or slept in a shelter? | | 53 |  | 129 |  |  | **7.82 (2)** | **0.020** |
|  | Yes | 8 | 15.1% | 5 | 3.88% |  |  |  |
|  | No | 45 | 84.9% | 122 | 94.6% |  |  |  |
|  | Patient refused | 0 | 0.000% | 2 | 1.55% |  |  |  |
|  |  |  |  |  |  |  |  |  |
| Are you worried that the place you are living now is making you sick? | | 53 |  | 130 |  |  | 2.64 (2) | 0.267 |
|  | Yes | 6 | 11.3% | 12 | 9.23% |  |  |  |
|  | No | 47 | 88.7% | 112 | 86.2% |  |  |  |
|  | Patient refused | 0 | 0.000% | 6 | 4.62% |  |  |  |
|  |  |  |  |  |  |  |  |  |
| In the past 12 months, has lack of transportation kept you from medical appointments or medications? | | 61 |  | 157 |  |  | 0.403 (1) | 0.526 |
|  | Yes | 14 | 23.0% | 30 | 19.1% |  |  |  |
|  | No | 47 | 77.1% | 127 | 80.9% |  |  |  |
|  | Patient refused | 0 | 0.000% | 0 | 0.000% |  |  |  |
|  |  |  |  |  |  |  |  |  |
| *Patient characteristics* | |  |  |  |  |  |  |  |
| Age (in years), mean (SD) | | 61 | 49.4 (15.6) | 158 | 56.3 (13.4) | **-6.88 (2.11)** | **-3.25 (217)** | **0.001** |
|  |  |  |  |  |  |  |  |  |
| Gender | | 61 |  | 158 |  |  | 3.78 (1) | 0.052 |
|  | Male | 24 | 39.3% | 41 | 26.0% |  |  |  |
|  | Female | 37 | 60.7% | 117 | 74.1% |  |  |  |
|  |  |  |  |  |  |  |  |  |
| Race/ethnicity | | 61 |  | 158 |  |  | 3.26 (4) | 0.515 |
|  | Hispanic | 4 | 6.56% | 6 | 3.80% |  |  |  |
|  | Black/African American | 30 | 49.2% | 82 | 51.9% |  |  |  |
|  | Other/more than one race^‡^ | 0 | 0.000% | 5 | 3.16% |  |  |  |
|  | Patient refused/unknown | 0 | 0.000% | 1 | 0.633% |  |  |  |
|  | White |  |  |  |  |  |  |  |
|  |  |  |  |  |  |  |  |  |
| Exercise duration per week (in minutes), mean (SD) | | 52 | 11.6 (11.7) | 128 | 10.9 (11.6) | 0.710 (1.91) | 0.371 (178) | 0.711 |
|  |  |  |  |  |  |  |  |  |
| Preferred language | | 61 |  | 158 |  |  | 0.388 (1) | 0.533 |
|  | Other^§^ | 0 | 0.000% | 1 | 0.633% |  |  |  |
|  | English | 61 | 100% | 157 | 99.4% |  |  |  |
|  |  |  |  |  |  |  |  |  |
| Primary payer | | 61 |  | 158 |  |  | 9.42 (5) | 0.093 |
|  | Private/commercial^\|\|^ | 17 | 27.9% | 51 | 32.3% |  |  |  |
|  | Managed Care | 13 | 21.3% | 21 | 13.3% |  |  |  |
|  | Medicaid^¶^ | 10 | 16.4% | 23 | 14.6% |  |  |  |
|  | Medicare | 5 | 8.20% | 13 | 8.23% |  |  |  |
|  | Medicare Advantage | 4 | 6.56% | 32 | 20.3% |  |  |  |
|  | Others^#^ | 12 | 19.7% | 18 | 11.4% |  |  |  |
|  |  |  |  |  |  |  |  |  |
| Presence of comorbidities | | 61 |  | 158 |  |  |  |  |
|  | Cancer | 7 | 11.5% | 16 | 10.1% |  | 0.085 (1) | 0.770 |
|  | COPD | 5 | 8.20% | 22 | 13.9% |  | 1.34 (1) | 0.248 |
|  | CHF | 6 | 9.84% | 16 | 10.1% |  | 0.004 (1) | 0.949 |
|  | CVD | 5 | 8.20% | 13 | 8.23% |  | 0.000 (1) | 0.994 |
|  | Mental health disorder | 21 | 34.4% | 70 | 44.3% |  | 1.77 (1) | 0.184 |
|  | Asthma | 7 | 11.5% | 31 | 19.6% |  | 2.04 (1) | 0.154 |
|  | Alzheimer’s | 0 | 0.000% | 0 | 0.000% |  |  |  |
|  | ETOH abuse | 5 | 8.20% | 4 | 2.53% |  | 3.58 (1) | 0.058 |
|  | Drug abuse | 16 | 26.2% | 27 | 17.1% |  | 2.33 (1) | 0.127 |

*Mean (SD) provided for continuous variables

^†^Comparison tests include independent sample t-test (for continuous variables), McNemar’s chi-squared test (for binary variables), and Extended Mantel-Haenszel (Cochran-Mantel-Haenszel) Stratified Test of Association (for categorical variables with more than 2 categories). McNemar’s chi-squared test is a special case of Cochran-Mantel-Haenszel test. Differences are statistically significant if p<0.05

^‡^Other/more than one race includes American Indian or Alaska Native, Native Hawaiian or Other Pacific Islander, Asian, and individuals with two or more races

^§^Other language includes Spanish and American Sign Language

^||^Private/commercial includes Bluecross Blueshield and Commercial

^¶^Medicaid includes Medicaid and Medicaid MCO

^#^Other payers include other, self-pay, pending Medicaid, and Tricare

**Methods A.1: Little’s test for data missing completely at random and multiple imputation**

We used Little’s test for data missing completely at random to examine whether missingness in the clinical outcome variables was independent of observed and unobserved variables. In tests for clinical outcomes at baseline and follow-up, except HbA1c, results had p<0.05 for all outcomes (appendix Table A.4). This indicates that baseline outcomes do not fully explain missingness at follow-up. We subsequently performed the test for baseline clinical outcomes and observed patient characteristics (age, gender, race/ethnicity, language, primary payer, and comorbidities), and found that the latter accounting for missingness at follow-up. Given these results, multiple imputation was used to impute missing clinical outcomes data at both baseline and follow-up.

Baseline HbA1c, BP, and BMI were imputed using chained (sequential) model and 10 imputation sets with patient characteristics as independent variables. Follow-up clinical outcomes were imputed similarly with baseline outcomes as an additional independent variable.

**Table A.4: Results from Little’s missing completely at random test for clinical outcomes**

| **Dependent variables** | **n** | $\boldsymbol{\chi}^{\boldsymbol{2}}$ **(df)** | **p** |
| --- | --- | --- | --- |
| HbA1c at baseline and six months follow-up | 197 | 1.38 (4) | 0.848 |
| Systolic blood pressure at baseline and six months follow-up | 398 | 8.36 (2) | 0.015 |
| Diastolic blood pressure at baseline and six months follow-up | 398 | 7.16 (2) | 0.028 |
| BMI at baseline and six months follow-up | 436 | 8.90 (2) | 0.012 |
| HbA1c at baseline and patient characteristics^a^ | 218 | 39.5 (28) | 0.073 |
| BMI at baseline and patient characteristics^a^ | 438 | 18.9 (28) | 0.901 |

^a^Patient characteristics include age, gender, race/ethnicity, preferred language, primary payer, and presence of each comorbidity. Results were statistically significant if p<0.05

**Table A.5: Clinical outcomes and healthcare charges for intervention and control group at baseline and six months follow-up**

| **Outcomes** | | **n** | **Mean (SD)** | | **Mean diff.**  **(SE)** | **t** | **p** |
| --- | --- | --- | --- | --- | --- | --- | --- |
|  |  |  | **Baseline** | **6 months** |  |  |  |
| HbA1c (%) | |  |  |  |  |  |  |
|  | Intervention | 109 | 7.72 (2.21) | 7.42 (1.62) | -0.297 (0.193) | -1.54 | 0.131 |
|  | Control | 109 | 7.28 (1.73) | 7.12 (1.59) | -0.160 (0.175) | -0.910 | 0.364 |
|  |  |  |  |  |  |  |  |
| Systolic BP (mmHg) | |  |  |  |  |  |  |
|  | Intervention | 199 | 134.6 (19.2) | 129.6 (16.4) | -5.00 (1.55) | -3.22 | 0.002 |
|  | Control | 199 | 131.3 (17.5) | 129.8 (17.5) | -1.51 (1.63) | -0.920 | 0.360 |
|  |  |  |  |  |  |  |  |
| Diastolic BP (mmHg) | |  |  |  |  |  |  |
|  | Intervention | 199 | 81.6 (14.3) | 80.1 (11.0) | -1.56 (0.930) | -1.67 | 0.095 |
|  | Control | 199 | 80.8 (11.3) | 79.9 (10.6) | -0.892 (0.949) | -0.940 | 0.484 |
|  |  |  |  |  |  |  |  |
| BMI | |  |  |  |  |  |  |
|  | Intervention | 219 | 35.2 (9.72) | 35.2 (9.56) | 0.027 (0.180) | 0.150 | 0.880 |
|  | Control | 219 | 35.9 (10.5) | 36.0 (10.5) | 0.099 (0.195) | 0.510 | 0.613 |
|  |  |  |  |  |  |  |  |
| PCP charges, 2023 USD | | | | | | | |
|  | Intervention | 212 | 387.6 (319.5) | 233.3 (377.9) | -154.3 (30.4) | -5.08 | 0.000 |
|  | Control | 212 | 682.8 (456.3) | 463.9 (426.8) | -218.9 (36.9) | -5.94 | 0.000 |
|  |  |  |  |  |  |  |  |
| IP charges, 2023 USD | | |  |  |  |  |  |
|  | Intervention | 212 | 5877.9 ( 18327.9) | 6332.0 (32401.6) | 454.1 (2260.7) | 0.201 | 0.841 |
|  | Control | 212 | 3782.4 (15774.4) | 1778.4 (8589.5) | -2004.0 (1177.0) | -1.70 | 0.090 |
|  |  |  |  |  |  |  |  |
| ED charges, 2023 USD | | |  |  |  |  |  |
|  | Intervention | 212 | 263.4 (571.6) | 204.5 (446.4) | -59.0 (42.4) | -1.39 | 0.167 |
|  | Control | 212 | 251.4 (628.4) | 159.4 (370.9) | -92.0 (39.2) | -2.35 | 0.020 |

**Table A.6: Six-month changes in clinical outcomes for patients in intervention and control group (full model)**

| **Variables** | | **(1)** | **(2)** | **(3)** | **(4)** |
| --- | --- | --- | --- | --- | --- |
|  |  | **HbA1c** | **SBP** | **DBP** | **BMI** |
|  |  | **Mean (SE)** | **Mean (SE)** | **Mean (SE)** | **Mean (SE)** |
| Group (ref. = matched control) | | | |  |  |
|  | Intervention | 0.317  (0.287) | 3.11  (1.82) | 0.614  (1.20) | 0.176  (0.888) |
| Time (ref. = base) | | |  |  |  |
|  | 6 months | -0.160  (0.161) | -1.51  (1.65) | -0.892  (0.924) | 0.099  (0.190) |
| Intervention $\times$ 6 months | | -0.134  (0.230) | -3.49  (2.20) | -0.664  (1.32) | -0.073  (0.267) |
| Demographics | | |  |  |  |
| Age (de-mean) | | -0.013  (0.010) | -0.028  (0.070) | -0.224**  (0.046) | -0.286**  (0.044) |
| Gender (ref. = female) | | |  |  |  |
|  | Male | 0.214  (0.276) | 5.72**  (1.80) | 1.60  (1.09) | -2.33*  (1.00) |
| Race/ethnicity (ref. = White) | | | |  |  |
|  | Black/African American | 0.253  (0.290) | 2.24  (1.65) | 1.70  (1.06) | 0.886  (0.933) |
|  | Hispanic | 0.413  (0.630) | 8.88  (5.60) | 0.823  (2.92) | -2.32  (1.96) |
|  | Other^†^/more than one race | -0.206  (0.596) | 0.134  (4.83) | -1.27  (3.67) | -3.35  (1.91) |
|  | Patient refused | 0.772  (0.584) | 5.77  (6.64) | 5.39**  (2.51) | 6.64*  (3.22) |
| Language (ref. = other^‡^) | | | |  |  |
|  | English | -0.230  (0.733) | 2.69  (4.88) | 3.52  (2.91) | 2.60  (2.51) |
| Payer (ref. = private/commercial^§^) | | | |  |  |
|  | Managed Care | -0.327  (0.352) | -2.11  (2.40) | -1.85  (1.47) | -0.194  (1.38) |
|  | Medicaid^\|\|^ | 0.142  (0.462) | 6.40*  (2.52) | 3.96*  (1.57) | -2.11  (1.33) |
|  | Medicare | -0.257  (0.483) | 2.88  (3.04) | -1.32  (1.63) | 1.00  (1.48) |
|  | Medicare Advantage | -0.619  (0.391) | 0.652  (2.53) | -2.42  (1.62) | 2.18  (1.40) |
|  | Others^¶^ | -0.644  (0.397) | 2.30  (2.37) | 1.71  (1.55) | -0.247  (1.61) |
| Presence of comorbidities (ref. = no) | | | | |  |
|  | Cancer | 0.040  (0.418) | 2.55  (3.03) | -2.99*  (1.49) | -1.71  (1.25) |
|  | COPD | 0.375  (0.432) | -3.15  (2.34) | -0.453  (1.42) | -0.020  (1.26) |
|  | CHF | 0.593  (0.401) | -2.20  (2.52) | -1.22  (1.40) | 3.66**  (1.32) |
|  | CVD | 0.184  (0.433) | 2.13  (2.69) | 0.758  (1.81) | -0.406  (1.14) |
|  | Mental health disorder | -0.220  (0.286) | -3.88*  (1.71) | -2.75**  (1.04) | -0.267  (9.26) |
|  | Asthma | 0.004  (0.346) | -1.71  (1.77) | -1.83  (1.15) | 6.09**  (1.38) |
|  | Alzheimer’s | 0.423  (0.751) | -31.9**  (3.71) | -17.9**  (2.42) | -2.15  (2.10) |
|  | ETOH abuse | -1.21*  (0.562) | -3.13  (3.84) | 2.00  (2.53) | -6.65**  (1.85) |
|  | Drug abuse | -0.203  (0.414) | 4.08  (2.71) | 0.452  (1.58) | -1.33  (1.22) |
| Intercept | | 7.63**  (0.836) | 125.6**  (5.30) | 77.7**  (3.25) | 33.0**  (2.80) |
| N | | 434 | 796 | 796 | 874 |
| F-test | | F (24, 435.7) = 0.64 | F (24, 11712.1) = 67.81 | F (24, 12646.4) = 149.08 | F (24, 236445.3) = 41.60 |
| Prob > F | | 0.907 | 0.000 | 0.000 | 0.000 |

Standard error in parenthesis

**p<0.01, *p<0.05

^†^Other includes American Indian or Alaska Native, Native Hawaiian or Other Pacific Islander, Asian, and individuals with two or more races

^‡^Other language includes Spanish and American Sign Language
^§^Private/commercial includes Bluecross Blueshield and Commercial
^||^Medicaid includes Medicaid and Medicaid MCO
^¶^Other payers include other, self-pay, pending Medicaid, and Tricare

**Table A.7: Two-part model depicting six-month changes in healthcare charges between intervention and control groups (full model)**

| **Variables** | | **(1)** | | **(2)** | | **(3)** | |
| --- | --- | --- | --- | --- | --- | --- | --- |
|  |  | **PCP charges** | | **ED charges** | | **IP charges** | |
|  |  | **First part^#^, OR** | **Second part**^††^**, Coeff.** | **First part^#^, OR** | **Second part**^††^**, Coeff.** | **First part^#^, OR** | **Second part**^‡‡^**, Coeff.** |
| Group (ref.= matched control) | | |  |  |  |  |  |
|  | Intervention | 0.101**  (0.059) | -0.425**  (0.063) | 1.66 (0.527) | -0.070  (0.138) | 1.68  (0.682) | -0.113  (0.224) |
|  |  |  |  |  |  |  |  |
| Time (ref. = base) | |  |  |  |  |  |  |
|  | 6 months | 0.033**  (0.020) | -0.105  (0.060) | 0.743  (0.187) | -0.348**  (0.099) | 0.681  (0.294) | -0.292  (0.387) |
|  |  |  |  |  |  |  |  |
| Intervention $\times$ 6 months | | 0.677  (0.388) | 0.435**  (0.099) | 0.700  (0.262) | 0.292  (0.156) | 0.974  (0.532) | 0.507  (0.429) |
|  | |  |  |  |  |  |  |
| Demographics | |  |  |  |  |  |  |
| Age (de-mean) | | 1.03  (0.015) | 0.010**  (0.002) | 0.953**  (0.011) | -0.007  (0.004) | 0.979  (0.012) | 0.011  (0.009) |
|  | |  |  |  |  |  |  |
| Gender (ref. = female) | |  |  |  |  |  |  |
|  | Male | 0.610  (0.218) | -0.039  (0.060) | 0.834  (0.257) | -0.024  (0.104) | 1.45  (0.490) | 0.002  (0.220) |
|  |  |  |  |  |  |  |  |
| Race/ethnicity (ref.= White) | | |  |  |  |  |  |
|  | Black/African American | 1.22  (0.404) | -0.094  (0.063) | 0.807  (0.212) | -0.154  (0.108) | 0.469*  (0.147) | 0.046  (0.204) |
|  | Hispanic | 0.467  (0.442) | -0.039  (0.167) | 0.552  (0.474) | 0.047  (0.271) | 0.678  (0.412) | 1.78**  (0.416) |
|  | Other^†^/more than one race | 5.53  (6.46) | -0.303*  (0.120) | 0.314  (0.212) | -0.405  (0.384) | 1.84  (1.61) | 0.675*  (0.297) |
|  | Patient refused | 2.68  (1.81) | 0.201  (0.219) | 0.693  (0.879) | -0.003  (0.160) | (Omitted) | (Omitted) |
|  |  |  |  |  |  |  |  |
| Language (ref. = other^‡^) | |  |  |  |  |  |  |
|  | English | 0.030**  (0.040) | 0.038  (0.190) | 0.903  (0.875) | 0.324  (0.261) | 0.709  (0.440) | 1.35**  (0.332) |
|  |  |  |  |  |  |  |  |
| Payer (ref. = private/commercial^§^) | | |  |  |  |  |  |
|  | Managed Care | 0.534  (0.249) | 0.154  (0.098) | 1.03  (0.454) | -0.242  (0.158) | 1.14  (0.773) | 0.395  (0.524) |
|  | Medicaid^\|\|^ | 5.00**  (2.65) | 0.063  (0.081) | 1.69  (0.693) | 0.203  (0.130) | 3.01*  (1.53) | 0.102  (0.353) |
|  | Medicare | 0.538  (0.309) | 0.138  (0.096) | 3.01*  (1.55) | -0.115  (0.179) | 2.60  (1.46) | 0.110  (0.417) |
|  | Medicare Advantage | 0.640  (0.340) | -0.051  (0.088) | 2.55*  (1.13) | 0.298  (0.180) | 3.88**  (1.95) | -0.229  (0.372) |
|  | Others^¶^ | 3.36*  (1.93) | 0.119  (0.087) | 1.81  (0.678) | 0.065  (0.133) | 3.92**  (1.82) | 0.182  (0.311) |
|  |  |  |  |  |  |  |  |
| Presence of comorbidities (ref. = no) | | |  |  |  |  |  |
|  | Cancer | 0.413  (0.218) | -0.001  (0.096) | 3.00**  (1.26) | -0.392**  (0.146) | 5.17**  (1.99) | 0.369  (0.274) |
|  | COPD | 1.06  (0.536) | -0.087  (0.094) | 0.875  (0.366) | 0.301  (0.175) | 0.644  (0.306) | -0.297  (0.254) |
|  | CHF | 0.931  (0.467) | -0.158  (0.096) | 1.55  (0.669) | 0.005  (0.167) | 2.49*  (0.925) | 0.285  (0.207) |
|  | CVD | 0.960  (0.513) | 0.129  (0.139) | 1.37  (0.576) | 0.274  (0.143) | 2.11*  (0.761) | 0.121  (0.215) |
|  | Mental health disorder | 1.49  (0.503) | 0.202**  (0.065) | 1.29  (0.344) | 0.222**  (0.083) | 1.37  (0.451) | 0.323  (0.222) |
|  | Asthma | 1.53  (0.705) | 0.127  (0.079) | 1.59  (0.563) | 0.169  (0.129) | 0.559  (0.259) | 0.010  (0.314) |
|  | Alzheimer’s | 0.449  (0.375) | -0.532**  (0.141) | 12.6**  (8.22) | 0.562*  (0.233) | 29.6**  (25.9) | -0.878  (0.562) |
|  | ETOH abuse | 4.29*  (2.96) | 0.024  (0.110) | 1.81  (0.894) | 0.034  (0.144) | 2.44  (1.49) | 0.111  (0.286) |
|  | Drug abuse | 1.03  (0.486) | -0.045  (0.081) | 0.774  (0.281) | -0.041  (0.128) | 0.740  (0.290) | -0.357  (0.259) |
|  |  |  |  |  |  |  |  |
| Intercept | | 3447.4**  (5869.0) | 6.43**  (0.208) | 0.151  (0.160) | 6.24**  (0.307) | 0.030**  (0.025) | 9.13**  (0.450) |
| N | | 846 | 617 | 846 | 240 | 840 | 71 |
| Wald $\chi^{2}$ | | $\chi^{2}$(24)= 57.7 | $\chi^{2}$(24)= 316.6 | $\chi^{2}$(24)= 89.2 | - | $\chi^{2}$(23)= 87.5 | - |
| Prob > $\chi^{2}$ | | 0.000 | 0.000 | 0.000 | - | 0.000 | - |

Standard error in parenthesis

**p<0.01, *p<0.05

^†^Other includes American Indian or Alaska Native, Native Hawaiian or Other Pacific Islander, Asian, and individuals with two or more races

^‡^Other language includes Spanish and American Sign Language
^§^Private/commercial includes Bluecross Blueshield and Commercial
^||^Medicaid includes Medicaid and Medicaid MCO
^¶^Other payers include other, self-pay, pending Medicaid, and Tricare

^#^Model includes all healthcare charges and is estimated using a logit model with clustered standard errors

^††^Model includes healthcare charges>0 and is estimated using generalized estimated equation with an inverse Gaussian distribution, log link function, and robust standard errors

^‡‡^Model includes healthcare charges>0 and is estimated using generalized estimated equation with a gamma distribution, log link function and robust standard errors

**Table A.8: Generalized negative binomial model predicting differences in rate of primary care visits at baseline and six-months follow-up between intervention and control groups**

| **Variables** | | **PCP visits** |
| --- | --- | --- |
|  |  | **IRR**  **(SE)** |
| **Group (ref. = matched control)** | |  |
|  | Intervention | 0.836  (0.043) |
|  | *p-value* | 0.000 |
|  | |  |
| **Time (ref. = baseline)** | |  |
|  | 6 months | 0.961  (0.030) |
|  | *p-value* | 0.201 |
|  | |  |
| **Intervention × 6 months** | | 1.11  (0.050) |
| *p-value* | | 0.039 |
|  |  |  |
| **Patient demographics** | |  |
| Age | | 1.00  (0.001) |
| *p-value* | | 0.925 |
|  |  |  |
| Gender (ref. = female) | |  |
|  | Male | 0.964  (0.037) |
|  | *p-value* | 0.330 |
|  |  |  |
| Race (ref. = Black/African American) | |  |
|  | White | 0.998  (0.034) |
|  | *p-value* | 0.946 |
|  |  |  |
|  | Other* | 1.00  (0.117) |
|  | *p-value* | 0.990 |
|  |  |  |
|  | Patient refused | 1.05  (0.208) |
|  | *p-value* | 0.822 |
|  |  |  |
|  | Hispanic | 1.03  (0.074) |
|  | *p-value* | 0.721 |
|  |  |  |
|  |  |  |
| Primary payer (ref. = Private/commercial^†^) | |  |
|  | Medicaid^‡^/Managed Care | 1.04  (0.043) |
|  | *p-value* | 0.358 |
|  |  |  |
|  | Medicare/Medicare Advantage | 0.986  (0.051) |
|  | *p-value* | 0.782 |
|  |  |  |
|  | Other^§^ | 1.12  (0.048) |
|  | *p-value* | 0.023 |
|  |  |  |
| Number of comorbidities (ref. = 1) | |  |
|  | 2 | 1.04  (0.050) |
|  | *p-value* | 0.508 |
|  |  |  |
|  | 3+ | 1.08  (0.047) |
|  | *p-value* | 0.085 |
|  |  |  |
|  |  |  |
| Intercept | | 0.575  (0.054) |
| *p-value* | | 0.000 |
| No. of observations | | 874 |
| Wald test (df) | | $x$^2^ (14) = 27.91 |
| Prob > $x$^2^ | | 0.015 |

Note: IRR=incidence rate ratio; PCP=primary care physician
^*^Other includes American Indian or Alaska Native, Native Hawaiian or Other Pacific Islander, Asian, and individuals with two or more races
^†^Private/commercial includes Bluecross Blueshield and Commercial
^‡^Medicaid includes Medicaid and Medicaid MCO
^§^Other payers include other, self-pay, pending Medicaid, and Tricare

**Table A.9: Distribution of Euroqol-5D-5L dimension responses at baseline, six- and twelve-month follow up**

| **Dimension** | **Baseline,**  **n (%)** | **6-months follow up, n (%)** | **12-months follow up, n (%)** | **P^*^** |
| --- | --- | --- | --- | --- |
| ***Mobility*** | **217** | **160** | **66** | **0.749** |
| No problems | 130 (59.9) | 94 (58.8) | 33 (50) |  |
| Slight problems | 46 (21.2) | 40 (25.0) | 22 (33.3) |  |
| Moderate problems | 27 (12.4) | 17 (10.6) | 9 (13.6) |  |
| Severe problems | 10 (4.61) | 6 (3.75) | 1 (1.52) |  |
| Unable to walk | 4 (1.84) | 3 (1.88) | 1 (1.52) |  |
|  |  |  |  |  |
| ***Self care*** | **217** | **159** | **66** | **0.108** |
| No problems | 175 (80.7) | 136 (85.5) | 49 (74.2) |  |
| Slight problems | 28 (12.9) | 16 (10.1) | 12 (18.2) |  |
| Moderate problems | 11 (5.07) | 4 (2.52) | 5 (7.58) |  |
| Severe problems | 2 (0.922) | 2 (1.26) | 0 (0.000) |  |
| Unable to wash or dress | 1 (0.461) | 1 (0.629) | 0 (0.000) |  |
|  |  |  |  |  |
| ***Usual activities*** | **217** | **160** | **66** | **0.921** |
| No problems | 88 (40.6) | 76 (47.5) | 29 (43.9) |  |
| Slight problems | 78 (35.9) | 42 (26.3) | 18 (27.3) |  |
| Moderate problems | 38 (17.5) | 30 (18.8) | 17 (25.8) |  |
| Severe problems | 11 (5.07) | 9 (5.63) | 1 (1.52) |  |
| Unable to do usual activities | 2 (0.922) | 3 (1.88) | 1 (1.52) |  |
|  |  |  |  |  |
| ***Pain/discomfort*** | **217** | **159** | **66** | **0.617** |
| No pain/discomfort | 54 (24.9) | 40 (25.2) | 16 (24.2) |  |
| Slight pain/discomfort | 64 (29.5) | 51 (32.1) | 19 (28.8) |  |
| Moderate pain/discomfort | 57 (26.3) | 50 (31.4) | 27 (40.9) |  |
| Severe pain/discomfort | 28 (12.9) | 13 (8.18) | 2 (3.03) |  |
| Extreme pain/discomfort | 14 (6.45) | 5 (3.14) | 2 (3.03) |  |
|  |  |  |  |  |
| ***Anxiety/depression*** | **217** | **160** | **66** | **0.014** |
| Not anxious/depressed | 65 (30.0) | 56 (35.0) | 18 (27.3) |  |
| Slightly anxious/depressed | 67 (30.9) | 61 (38.1) | 24 (36.4) |  |
| Moderately anxious/depressed | 49 (22.6) | 31 (19.4) | 20 (30.3) |  |
| Severely anxious/depressed | 24 (11.1) | 7 (4.38) | 4 (6.06) |  |
| Extremely anxious/depressed | 12 (5.53) | 5 (3.13) | 0 (0.000) |  |

^*^Differences between baseline and follow up scores for each dimension were examined using the Skillings-Mack test. This non-parametric test examines differences in repeated measures data and is particularly suited for handling missing data in longitudinal assessments.
